# The global distribution and evolutionary history of the pT26‐2 archaeal plasmid family

**DOI:** 10.1111/1462-2920.14800

**Published:** 2019-10-21

**Authors:** Catherine Badel, Gaël Erauso, Annika L. Gomez, Ryan Catchpole, Mathieu Gonnet, Jacques Oberto, Patrick Forterre, Violette Da Cunha

**Affiliations:** ^1^ Institute for Integrative Biology of the Cell (I2BC) CEA, CNRS, Univ. Paris‐Sud, Université Paris‐Saclay Paris France; ^2^ Laboratoire de Microbiologie des Environnements Extrêmes (LM2E) Université de Bretagne Occidentale (UBO, UEB), Institut Universitaire Européen de la Mer (IUEM) – UMR 6197 Plouzané France; ^3^ Aix‐Marseille Université, CNRS/INSU, Université de Toulon, IRD, Mediterranean Institute of Oceanography (MIO) UM 110 Marseille France; ^4^ Département de Microbiologie Institut Pasteur, Unité de Biologie Moléculaire du Gène chez les Extrêmophiles (BMGE) Paris France

## Abstract

Although plasmids play an important role in biological evolution, the number of plasmid families well‐characterized in terms of geographical distribution and evolution remains limited, especially in archaea. Here, we describe the first systematic study of an archaeal plasmid family, the pT26‐2 plasmid family. The in‐depth analysis of the distribution, biogeography and host–plasmid co‐evolution patterns of 26 integrated and 3 extrachromosomal plasmids of this plasmid family shows that they are widespread in Thermococcales and Methanococcales isolated from around the globe but are restricted to these two orders. All members of the family share seven core genes but employ different integration and replication strategies. Phylogenetic analysis of the core genes and CRISPR spacer distribution suggests that plasmids of the pT26‐2 family evolved with their hosts independently in Thermococcales and Methanococcales, despite these hosts exhibiting similar geographic distribution. Remarkably, core genes are conserved even in integrated plasmids that have lost replication genes and/or replication origins suggesting that they may be beneficial for their hosts. We hypothesize that the core proteins encode for a novel type of DNA/protein transfer mechanism, explaining the widespread oceanic distribution of the pT26‐2 plasmid family.

## Introduction

Mobile genetic elements (MGEs) are a crucial component of the living world, being the major vehicles for horizontal gene transfer (HGT; Koonin and Wolf, 2008), agents of genomic recombination (Cossu *et al*., 2017) and cradles of novel genes (Keller *et al*., 2009; Forterre and Gaïa, 2016; Legendre *et al*., 2018). Whereas archaea are much more closely related to eukaryotes than to bacteria in terms of fundamental molecular mechanisms (replication, transcription and translation), the set of MGEs (mobilome) infecting archaea and bacteria are strikingly similar and very different from those present in eukaryotes (Forterre, 2013; Forterre *et al*., 2014). It is unclear if the observed resemblance between the archaeal and bacterial mobilomes is a result of convergence due to the comparable chromosome structure and organization of archaeal and bacterial cells, or if it reflects widespread distribution of these MGEs by HGT between these two domains or perhaps inheritance of a similar type of MGE present in the Last Universal Common Ancestor. Further studies on the archaeal mobilome may help to resolve this conundrum.

Most research on the archaeal mobilome has focused on a narrow range of model organisms, including Sulfolobales, Haloarchaeales, Thermococcales and a few methanogens. Among them, plasmids and viruses from the order Thermococcales (composed of the genera *Thermococcus*, *Pyrococcus* and *Palaeococcus*) represent some of the most hyperthermophilic MGEs known to date and have been studied in several laboratories (Forterre *et al*., 2014; Lossouarn *et al*., 2015; Wang *et al*., 2015). Screening of extrachromosomal MGEs in 190 Thermococcales strains showed that 40% of the tested strains carry at least one MGE (Prieur *et al*., 2004). Two viruses, PAV1 from *Pyrococcus abyssi* (Geslin *et al*., 2007) and TPV1 from *Thermococcus prieurii* (Gorlas *et al*., 2012), and 19 plasmids have been isolated and sequenced (Forterre *et al*., 2014; Lossouarn *et al*., 2015; Wang *et al*., 2015) and for seven their DNA replication proteins have been characterized biochemically (Marsin and Forterre, 1998, 1999; Soler *et al*., 2007; Béguin *et al*., 2014; Gill *et al*., 2014). Additionally, other MGEs have been detected in the course of genome sequencing projects, either as extrachromosomal or integrated plasmids (Fukui *et al*., 2005; Zivanovic *et al*., 2009; Vannier *et al*., 2011).

In Thermococcales, plasmids have been grouped into seven families based on their replication proteins (Table 1; Forterre *et al*., 2014). They will be named hereafter according to their prototype plasmids pGT5, pTN2, pT26‐2, pTBMP1, pAMT11, pTP2 and pTN3 (Erauso *et al*., 1996; Geslin *et al*., 2007; Soler *et al*., 2011; 2010; Vannier *et al*., 2011; Gonnet *et al*., 2011; Gorlas *et al*., 2013; Forterre *et al*., 2014; Gaudin *et al*., 2014; Gill *et al*., 2014; Lossouarn *et al*., 2015; Kazlauskas *et al*., 2018). The two families with pGT5 and pTP2 as prototypes correspond to small rolling‐circle replication plasmids (Erauso *et al*., 1996; Gorlas *et al*., 2013). These two plasmid families have been used to construct *E*. *coli–Thermococcus* shuttle vectors for genetic manipulation of Thermococcales (Lucas *et al*., 2002; Santangelo *et al*., 2008; Catchpole *et al*., 2018). The five other plasmid families probably replicate via a theta mode, as most of them encode DNA primases and/or helicases (Soler *et al*., 2010; Krupovic *et al*., 2013; Béguin *et al*., 2014; Forterre *et al*., 2014; Gill *et al*., 2014).

**Table 1:** List of Thermococcales plasmid families.

Plasmid family (type plasmid)	Replication mode	Related MGE	Size	References
pTN2	θ	PAV1	8.5–13 kb	Geslin *et al*. (2007), Soler *et al*. (2010), Krupovic *et al*. (2013), Gill *et al*. (2014), and Kazlauskas *et al*. (2018)
pTBMP1	θ	–	55.5 kb	Vannier *et al*. (2011)
pAMT11	θ	TKV1	18.3–20.5 kb	Gonnet *et al*. (2011)
pT26‐2	θ	TKV2, TKV3	17–38 kb	Soler *et al*. (2010)
pTN3	θ	TKV4	13.8–20.2 kb	Gonnet *et al*. (2011), Gaudin *et al*. (2014), and Cossu *et al*. (2017)
pGT5	RC	–	3.4 kb	Erauso *et al*. (1996)
pTP2	RC	–	2 kb	Gorlas *et al*. (2013)

θ, theta mode; c, rolling circle.

The prototype plasmid of the pT26‐2 family was isolated from *Thermococcus* sp. 26‐2 collected in the East Pacific Ocean (Lepage *et al*., 2004). Later, eight plasmids of the pT26‐2 family were identified as integrated elements in the genomes of Thermococcales and Methanococcales (Soler *et al*., 2010) as well as a free plasmid, pMEFER01, in the hyperthermophile *Methanocaldococcus fervens* (Soler *et al*., 2011). The pT26‐2 plasmid family is composed of mid‐sized plasmids ranging from 17 to 38 kb, which encode many transmembrane proteins and an AAA+ ATPase hypothesized to be involved in DNA transfer (Soler *et al*., 2010). Comparative analysis suggested that their sequences can be divided into two regions: a highly conserved region of 12 genes, which includes 7 core genes present in all 10 plasmids of the family known at that time (t26‐5p, 6p, 7p, 11p, 13p, 14p and 15p), and a variable region that includes singleton ORFans (open reading frames without matches in current sequence databases) and genes of various origins (Soler *et al*., 2010). The structure of one of the two largest core proteins was determined (t26‐6p; Keller *et al*., 2009) and this protein contains several domains exhibiting novel folds, supporting the idea that plasmids and/or viruses could be reservoirs of novel protein folds (Keller *et al*., 2009; Soler *et al*., 2010). The core proteins encoded by the prototype plasmid pT26‐2 had homologues only in related elements, raising challenging questions concerning the origin of this family.

Members of the pTN2 plasmid family were also reported in both Thermococcales and Methanococcales (Krupovic *et al*., 2013). The presence of related plasmids in these two archaeal orders could be explained by the fact that theses archaea can share similar biotopes. The Thermococcales are strictly anaerobic hyperthermophiles (optimal growth temperature > 80°C) that are ubiquitous in hydrothermal vent systems (Zillig *et al*., 1983; Fiala and Stetter, 1986; Takai *et al*., 2001). The majority of Thermococcales were isolated from marine geothermal environments, both shallow and deep hydrothermal vents, and a few strains were also isolated from continental oil reservoirs (at high temperature and salinity; Ravin *et al*., 2009) and from freshwater terrestrial hot springs (Antranikian *et al*., 2017). The Methanococcales are also strictly anaerobic, but, in contrast to Thermococcales, they are not restricted to high‐temperature environments; *Methanocaldococcaceae* (*Methanocaldococcus* and *Methanotorris*) are hyperthermophiles and *Methanococcaceae* (*Methanococcus* and *Methanothermococcus*) are either hyperthermophiles or mesophiles (Supporting Information Fig. S1). All members of these two families were isolated from aquatic environments and are capable of forming methane by reduction of CO_2_ with H_2_ (Albers and Siebers, 2014).

The presence of related plasmids in Thermococcales and Methanococcales could be also explained by the fact that these two archaeal orders are phylogenetically closely related. They both belong to the Euryarchaeota cluster I sensu Raymann *et al*. (containing Thermococcales, Methanococcales, Methanopyrales and Methanobacteriales; Brochier‐Armanet *et al*., 2011; Raymann *et al*., 2015) and even form sister groups in some analyses (Makarova *et al*., 2015). However, most analyses support the super‐class Methanomada, which groups Methanococcales with other group I methanogens, i.e., Methanobacteriales and Methanopyrales (Supporting Information Fig. S1; Adam *et al*., 2017; Da Cunha *et al*., 2017).

Compared with the 100 archaeal genomes available in 2010 (Adam *et al*., 2017), several genome and metagenome sequencing projects have increased the number of available archaeal assemblies to 1,883 (November 2017) and to 3,541 (June 2019), including genomes of Thermococcales, Methanococcales and other Euryarchaeota. In particular, metagenomic analyses led to the identification of two new candidate archaeal orders, the Methanofastidiosa and the Theionarchaea (Nobu *et al*., 2016; Lazar *et al*., 2017), that branch as sister groups to Thermococcales in archaeal phyogenies, forming the super‐class Acherontia (Adam *et al*., 2017) (Supporting Information Fig. S1). These new orders are thus good candidates to detect MGE related to those of Thermococcales. This prompted us to update the search for plasmids of the pT26‐2 family in the hope of expanding the number of known elements, which could shed light on their origins, functions and mechanism of transfer. Here, we report the identification by genome data mining of 16 new members and the isolation of the first plasmid of this family from a *Pyrococcus* species. Despite the increase in the number of archaeal and bacterial genomes now available, the presence of plasmids of the pT26‐2 family remain limited to Thermococcales and Methanococcales. These plasmids use different types of replication proteins and a set of conserved core proteins that could provide some selective advantage to their hosts following integration. Plasmids present in Thermococcales and Methanococcales form two well‐defined monophyletic groups and exhibit different integration strategies mediated by non‐orthologous types of integrases. Our different phylogenetic analyses suggest that recent transfers of plasmids from the pT26‐2 family have occurred between different Thermococcales but not between the Thermococcales and Methanococcales, despite their hosts being often present in the same geographic locations. Although the origin of this plasmid family remains unknown, their modular structure and broad distribution across two archaeal orders sharing the same environment provides a unique opportunity to study plasmid evolution.

## Results

### *First free Pyrococcus plasmid and new integrated plasmid of the pT26‐2 family*

In order to expand the diversity of plasmids of the pT26‐2 family, each of the seven previously identified core genes (Soler *et al*., 2010) were used as query sequences to identify homologues in complete or partial archaeal, bacterial or eukaryotic genomes (see Methods section). Plasmids encoding these homologues were then characterized by genome context analysis. We identified 17 new plasmids of the pT26‐2 family integrated in Thermococcales and Methanococcales genomes, more than doubling the number of known elements (Table 2). In addition, we isolated and sequenced a new plasmid of this family, pGE2 (23,702 bp), from the strain GE2 belonging to the *P*. *abyssi* species (Erauso *et al*., 1993). pGE2 is the first plasmid of the pT26‐2 family isolated from *a Pyrococcus* species. In primary cultures of GE2 strain, pGE2 coexisted both as free and integrated copies, but the free copy was lost during subculturing (see Methods section for more information). Recently, the whole genome of *P*. *abyssi* strain GE2 was sequenced in the framework of a large project on comparative genomics of Thermococcales, and we were only able to detect the integrated plasmid from the assembly.

**Table 2:** List of plasmids of the pT26‐2 family.

Element	Host	Integration location	Att length	State	Access	Reference
pT26‐2	*Themococcus* sp. 26–2	1–21,566	51	Free/integrated	295126597	Soler *et al*. (2010)
TKV2	*Thermococcus kodakarensis* KOD1	320,075–347,187	48/53	Integrated	AP006878.1	Keller *et al*. (2009)
TKV3	*Thermococcus kodakarensis* KOD1	499,284–526,865	48/53	Integrated	AP006878.1	Fukui *et al*. (2005)
TguDSM11113_IP1	*Thermococcus guayamensis* DSM11113	153,065–178,766	46	Integrated	CPU007140.1	This analysis
TliDSM5473_IP1	*Thermococcus litoralis* DSM 5473	500,722–523,246	44	Integrated	CP006670.1	This analysis
TbaCH5_IP1	*Thermococcus barophilus* CH5	770,185–788,746	44	Integrated	CP013050.1	This analysis
TbaCH5_IP2	*Thermococcus barophilus* CH5	2,013,643–2,038,136	48	Integrated	CP013050.1	This analysis
TspJCM11816_IP1	*Thermococcus* sp. JCM11816	162,578–186,018	49	Integrated	Ga0128353_102	This analysis
TGV1	*Thermococcus gammatolerans* EJ3	621,669–642,462	50	Integrated	CP001398.1	Keller *et al*. (2009)
TceDSM17994_IP1	*Thermococcus celericrescens* DSM17994	15,770–43,341	129	Integrated	NZ_LLYW01000013	This analysis
PchGC74_IP1	*Pyrococcus chitonophagus* GC74	1,137,169–1,159,084	46	Integrated	NZ_CP015193	This analysis
PkuNCB100_IP1	*Pyrorcoccus kukulkanii* sp. NCB100	456,321–486,708	102	Integrated	CP010835.1	This analysis
PHV1	*Pyrococcus horikoshii* OT3	1,061,525–1,083,228	47	Integrated	BA000001.2	Keller *et al*. (2009)
PyaCH1_IP16	*Pyrococcus yayanosii* CH1	1,238,312–1,255,830	46	Integrated	CP002779	This analysis
PspNA2_IP1	*Pyrococcus* sp. NA2	1,199,678–1,221,811	47	Integrated	CP002670	This analysis
pGE2 = PabGE2_IP1	*Pyrococcus abyssi* GE2	1,467,989–1,488,841	48	Free/integrated	–	This analysis
MMC6V1	*Methanococcus maripaludis* C6	358–48,565	56	Integrated	NC_009975	Keller *et al*. (2009)
MMC7V1	*Methanococcus maripaludis* C7*	No detectable limits	–	Integrated	NC_009637	Keller *et al*. (2009)
MMC7V2	*Methanococcus maripaludis* C7	1,436,513–1,469,347	56	Integrated	NC_009637	Keller *et al*. (2009)
MMPV1 = MmaS2_IP	*Methanococcus maripaludis* S2	735,195–773,477	53	Integrated	NC_005791	Keller *et al*. (2009)
MmaKA1_IP1	*Methanococcus maripaludis* KA1	466,296–491,741	54	Integrated	AP011526	This analysis
MmaOS7_IP1	*Methanococcus maripaludis* OS7	45,126–475,878	54	Integrated	AP011528	This analysis
MmaC5_IP1	*Methanococcus maripaludis* C5*	No detectable limits	–	Remnant		This analysis
MmaX1_IP1	*Methanococcus maripaludis* X1	No detectable limits	–	Integrated	340623184	This analysis
MVV1	*Methanococcus voltae* A3	1,715,487–1,742,050	102	Integrated	NC_014222	Keller *et al*. (2009)
MthDSM2095_IP1	*Methanothermococcus thermolithotrophicus* DSM2095	1–21,165	54	–	NZ_AQXV01000029	This analysis
MigKol5_IP1	*Methanotorris igneus* Kol5	500,181–524,602	58	Integrated	NC_015562	This analysis
MspFS406‐22_IP1	*Methanocaldococcus* sp. FS406‐22	1,092,561–1,123,012	54	Integrated	NC_013887	This analysis
pMEFER01	*Methanocaldococcus* fervens AG86	1–22,190	57	Free	NC_013157	Soler *et al*. (2011)

**Figure 1:**
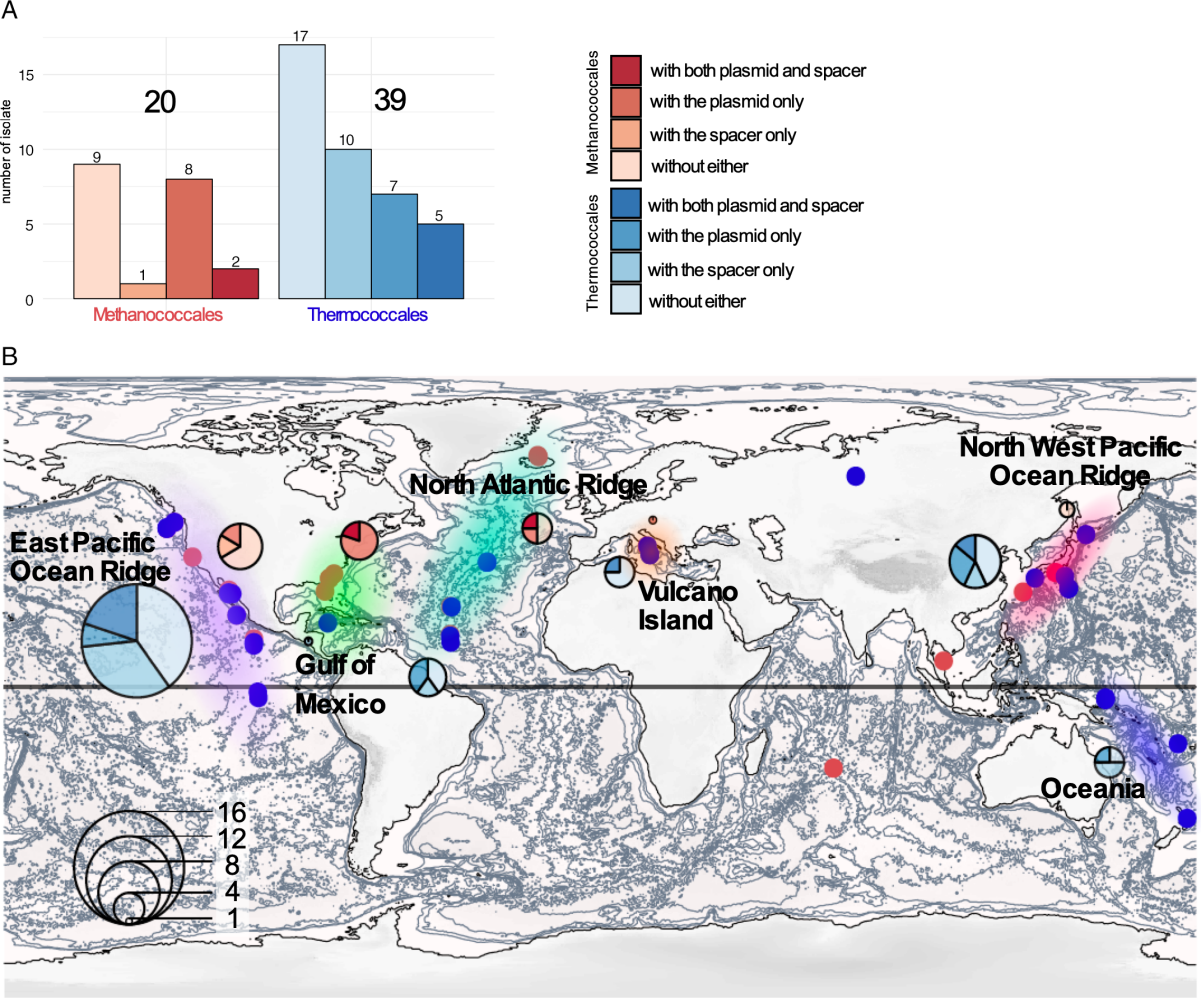
Biogeography of the Thermococcales and Methanococcales isolation sites of the NCBI available genomes. A. Barplot indicating the number of Methanococcales and Thermococcales isolates. For both orders, we also indicate the number of isolate containing a spacer against a plasmids of the pT26‐2 family, or containing a plasmids of the pT26‐2 family t or containing both. B. The isolation sites corresponding to Methanococcales and Thermococcales are indicated on the world map by red and blue dots respectively. Six major regions have been also indicated by different cloud on the world map *East Pacific Ocean Ridge*, *Gulf of Mexico*, *North Atlantic Ridge*, *Vulcano island*, *North West Pacific Ocean Ridges* and *Oceania*. For each region, the number of isolate is indicated with a pie chart and the presence of plasmids of the pT26‐2 family or a spacer against theses are indicated using the same colour code than in the a panel. [Color figure can be viewed at http://wileyonlinelibrary.com]

We could not find members of the pT26‐2 family in any genome outside of the Thermococcales and Methanococcales orders, despite the recent discovery of several new archaeal lineages closely related to these species, such as the Methanofastidiosa and the Theionarchaea. The 29 members of the pT26‐2 family are widespread within the two orders with 30% (12 of 39) of available Thermococcales genomes and 45% (10 of 22) of available Methanococcales genomes containing at least one of them (Fig. 1A). One *Methanococcus* and two *Thermococcus* strains contain two integrated plasmids. In the case of *Thermococcus kodakarensis*, the attachment sites (att sites) of the plasmids of the pT26‐2 family (TKV2 and TKV3), are not identical and are unexpectedly mixed (Supporting Information Fig. S2). This can be explained by an inversion between the two integrated plasmids (Supporting Information Fig. S2). Interestingly, a back‐inversion between TKV2 and TKV3 was previously detected experimentally in a sub‐population (<10%) of *T*. *kodakarensis* TS559 cells (Gehring *et al*., 2017), which restored their excision potentiality. This back‐inversion was asymmetrical and led to the gain or loss of 2 kb (four ORFans) in TKV3 and TKV2 respectively. In this sub‐population, the mixed plasmid of the pT26‐2 family has the potential to be mobile.

In order to identify strains which likely encountered plasmids of the pT26‐2 family in the past but no longer encode them, we searched for CRISPR spacers against these plasmids in the CRISPRdb database (Grissa *et al*., 2007). We only found such spacers in the genomes of Thermococcales and Methanococcales, confirming that plasmids of the pT26‐2 family have a restricted host range (Fig. 1A). Notably, we did not detect any CRISPR spacers against these plasmids in the MAGs of *Methanofastidiosa* and *Theionarchaea*, which are sister groups of Thermococcales (Adam *et al*., 2017). Among the available genomes, 30% of the Thermococcales and 15% of the Methanococcales genomes contain a CRISPR spacer against plasmids of the pT26‐2 family. When combined with the data on plasmid distribution, 56% of Thermococcales and 55% of Methanococcales have either a resident plasmid of the pT26‐2 family or a CRISPR spacer against them (Fig. 1A), indicating that more than half of Thermococcales and Methanococcales strains have encountered a plasmid of this family at least once during their evolution. In addition, we observed that 5 of the 12 Thermococcales isolates containing a plasmid of the pT26‐2 family also contain a spacer against a different plasmid of the same family. For example, in the genomes of *T*. *kodakarensis* and *Thermococcus guaymasensis*, the CRISPR loci contain spacers against the plasmids TliDSM11113_IP1 and TceDSM17994_IP1, which are found in integrated form in the genomes of *Thermococcus litoralis* and *Thermococcus celericresens* respectively. We were unable to find in Thermococcales genomes CRISPR spacers against plasmids of the pT26‐2 family integrated in the genomes of Methanococcales and vice versa. This suggests that individual members of this family are not able to colonize hosts from both taxonomic orders. Together, these results suggest that plasmids of the pT26‐2 family are widespread and mobile within Thermococcales and Methanococcales but remain limited to these two orders.

### *Plasmids of the pT26‐2 family are geographically widespread*

We compared the geographic distribution of hosts harbouring plasmids from the pT26‐2 family to those of all Methanococcales and Thermococcales isolates whose genomes are available from NCBI (Fig. 1B). Such comparative analysis allowed us to estimate the impact of possible isolation bias in the pT26‐2 plasmid family distribution in six major geographical areas. Except for a handful of *Thermococcus* species isolated from terrestrial host–springs, most strains were isolated from various marine environments, particularly deep‐sea hydrothermal vents located along oceanic ridges (in the Atlantic, Pacific, and Indian Oceans) or from volcanic back‐arcs in the Mediterranean Sea. The available genomes originate mostly from strains isolated in the northern hemisphere. In addition, the six *Methanococcus maripaludis* strains (out of seven) whose genomes contains a plasmid of the pT26‐2 family were isolated from neighbouring sites in the Gulf of Mexico (light green region in the Fig. 1B), indicating that the relative abundance of these plasmids in Methanococcales could be overestimated. In several cases, *Thermococcus*, *Pyrococcus*, *Paleococcus*, *Methanocaldococcus* and *Methanotorris* strains were isolated from the same deep‐sea hydrothermal sites, such as the East Pacific Ocean ridge (Fig. 1B), confirming that archaea from these two orders can share the same habitat.

To analyse plasmid distribution, we mapped information about the presence of plasmids of the pT26‐2 family and the presence of CRISPR spacers corresponding to their sequences on our biogeographic analysis (Fig. 1B). This revealed that plasmids of the pT26‐2 family were in contact with Thermococcales or Methanococcales isolates from all the different sampling regions, containing the six major regions *East Pacific Ocean Ridge*, *Gulf of Mexico*, *North Atlantic Ridge*, *Vulcano Island*, *North West Pacific Ocean Ridges* and *Oceania* (Fig. 1). It is thus clear that plasmids of the pT26‐2 family are abundant and widespread in Thermococcales and Methanococcales all over the world.

### *No horizontal transfer observed between Thermococcales and Methanococcales*

**Figure 2:**
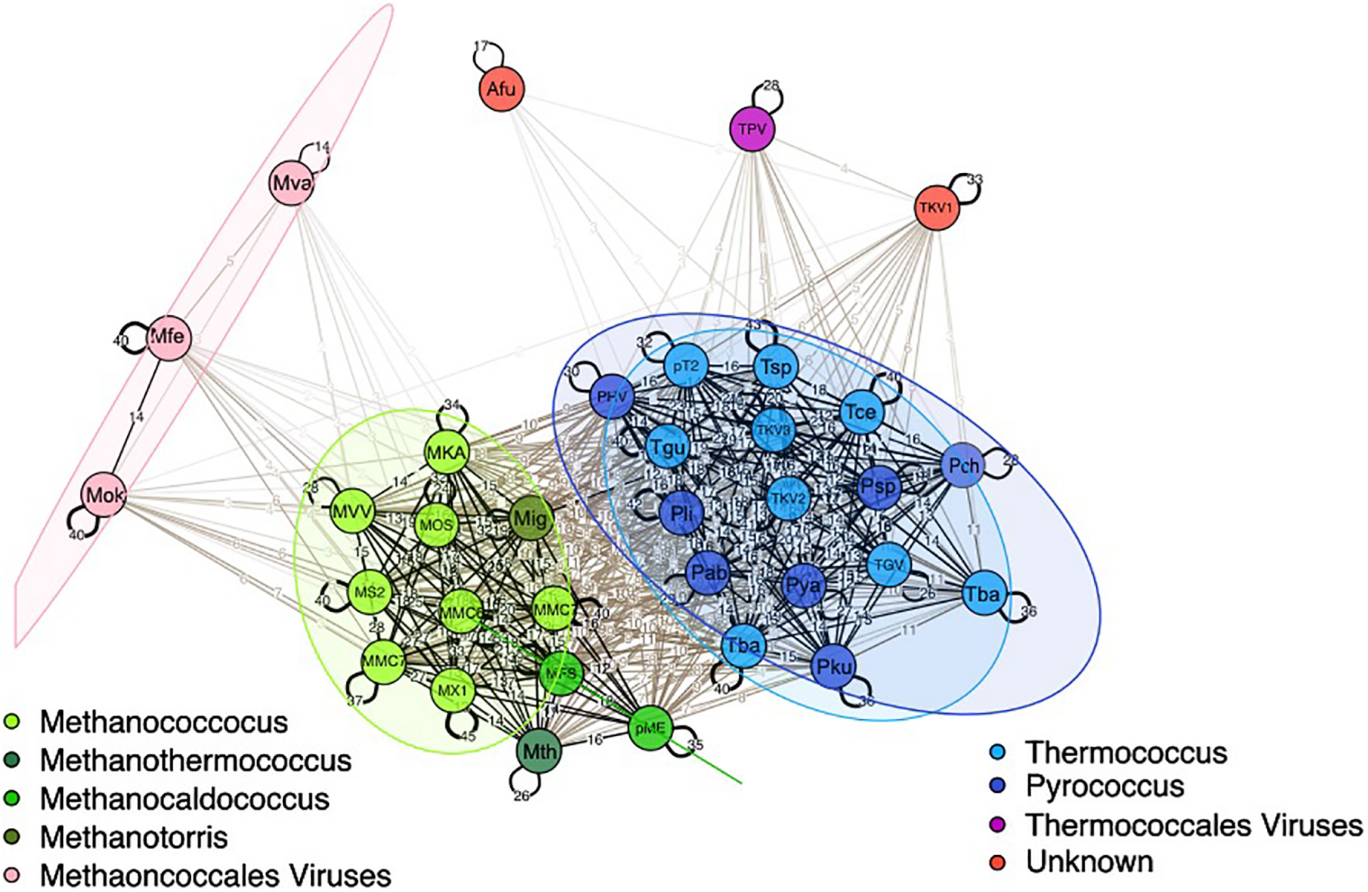
Network view of plasmids of the pT26‐2 family conservation. Results of Bidirectional best‐hit are represented as a network. The line thickness is related to the number of conserved genes between two elements. In addition for plasmids of the pT26‐2 family, they are coloured depending of their host genera in several kind of green for Methanococcales and two kind of blue for Thermococcales. This network analysis suggests that pT26‐2 and related elements are not transferred between the two orders and have co‐evolved with theirs host. This network show that some plasmids of the pT26‐2 family shared genes with archaeal viruses or other unknown kind of archaeal MGEs. [Color figure can be viewed at http://wileyonlinelibrary.com]

**Figure 3:**
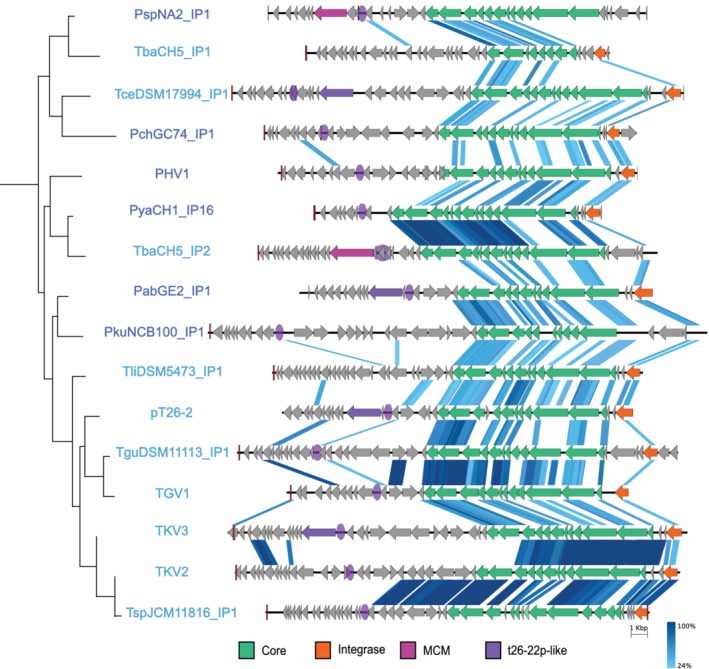
Comparison of Thermococcales plasmids of the pT26‐2 family. In this schematic representation, the CORE genes and the integrase genes are indicated in green and orange respectively. The different genes encoding for putative replication protein are indicated with different shade of purple. The result of conservation between two plasmids of the pT26‐2 family by tblastx is indicated with several shade of blue based on the protein identity percentage. The schematic phylogenetic tree in the left corresponds to a part of the phylogenetic tree obtained with the concatenation of the core proteins in Fig. 5. [Color figure can be viewed at http://wileyonlinelibrary.com]

**Figure 4:**
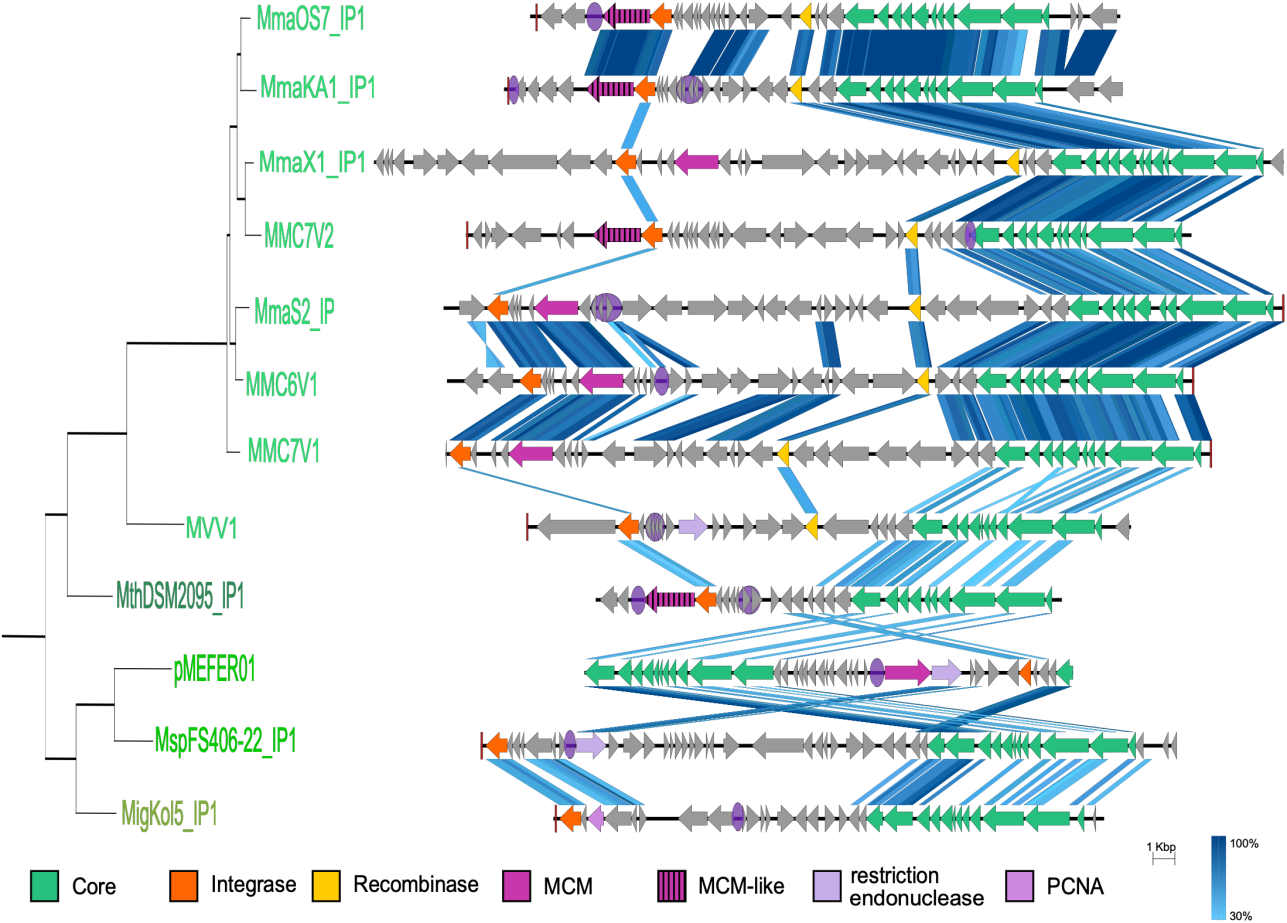
Comparison of Methanococcales plasmids of the pT26‐2 family. In this schematic representation, the CORE genes and the integrase genes are indicated in green and orange respectively. The location of the putative replication origin is indicated on the plasmid with a purple circle. The different genes encoding for putative replication protein are indicated with different shade of purple. The result of conservation between two plasmids of the pT26‐2 family by tblastx is indicated with several shade of blue based on the protein identity percentage. The schematic phylogenetic tree in the left corresponds to a part of the phylogenetic tree obtained with the concatenation of the core proteins in Fig. 5. [Color figure can be viewed at http://wileyonlinelibrary.com]

**Figure 5:**
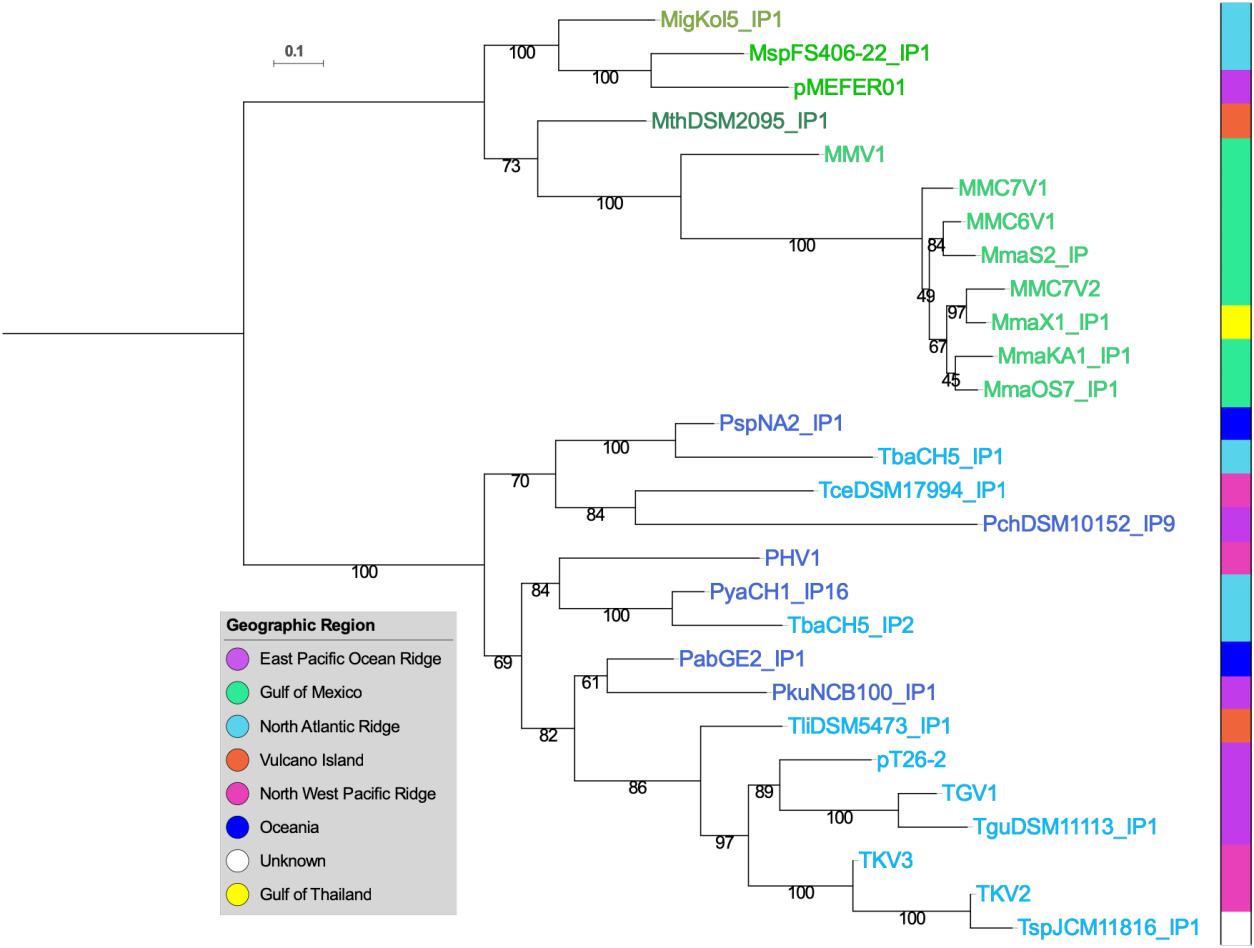
Maximum likelihood tree of the concatenated CORE proteins. The isolation region is indicated by a coloured square. The scale‐bars represent the average number of substitutions per site. Values at nodes represent support calculated by nonparametric bootstrap (out of 100). [Color figure can be viewed at http://wileyonlinelibrary.com]

The robustness of the previously predicted seven core genes in all 29 plasmids of the pT26‐2 family was shown using a variety of methods [Reciprocal Best Hit (RBH) and SiLiX (SIngle LInkage Clustering of Sequences) described in Methods section]. The comparative analysis of plasmids of the pT26‐2 family by RBH analysis (Fig. 2; Supporting Information Fig. S3) reveals the presence of two distinct sub‐groups, one containing the plasmids of thr pT26‐2 family identified in Thermococcales (Fig. 3) and the other those in Methanococcales (Fig. 4). This observation again suggests that, despite often sharing the same geographic location, plasmids of the pT26‐2 family have not been recently transferred between the two archaeal orders and have co‐evolved with theirs hosts. To further test this hypothesis, we compared the individual and the concatenated phylogenetic trees obtained with the seven core proteins. Both trees were congruent, with Thermococcales and the Methanococcales forming two separate monophyletic groups with internal phylogenies rather similar to the host phylogenies. In detail, we observed a clear co‐evolution of the plasmids of the pT26‐2 family within the Methanococcales (Fig. 5), but we also observed putative HGT between different genera of Thermococcales that are also seen in the network analysis (Fig. 2). In the phylogenetic tree based on concatenation of the seven core proteins, the two monophyletic groups of plasmids present in either Thermococcales or Methanococcales were separated by a long branch (Fig. 5), clearly indicating the absence of recent HGT between the two groups.

### *The highly conserved region encodes a putative secretion system*

Plasmids of the pT26‐2 family present a highly conserved region of 12 genes with conserved synteny (from *t26‐4p* to *t26‐15p*; Figs. 3 and 4), which includes the 7 core genes is present in all plasmids of the pT26‐2 family (Soler *et al*., 2010). The 12 proteins of the highly conserved region do not share significant similarity with any other proteins in public databases outside of the pT26‐2 family (as detectable by HMMER or BLASTP). Nine of these proteins contain at least one (and up to five) putative transmembrane helices. In some of these transmembrane proteins, additional domains were also detected, e.g., a SH3 domain in the protein t26‐13p, a carboxypeptidase regulatory domain in the protein t26‐5p and a carbohydrate binding domain in the protein t26‐10p. Notably, carbohydrate‐binding domains are present in some viral capsid proteins, allowing the recognition of host cellular surfaces (Krupovic and Koonin, 2017). Interestingly, Phyre‐2 analyses point to strong structural similarities between the t26‐14p‐like core protein and several ATPases from the AAA+ superfamily: the HerA‐like hexameric DNA translocase VirB4 of type IV secretion systems of conjugative plasmids, or the genome packaging ATPase B204 from the *Sulfolobus* Turreted Icosahedral Virus 2. The Phyre‐2 analysis of the t26‐6p protein, containing the five predicted transmembrane helices, showed a confident structural similarity with the bacterial colicin B protein. In bacteria, this protein is a cytotoxic protein that forms a pore in the bacterial membrane that depletes the electrochemical potential of the membrane and results in cell death. The analysis of the protein encoded by the high conserved region suggests that these proteins could be involved in the translocation of DNA through membranes.

### *Functional modules encoded by non‐core genes*

The SiLiX analysis of all proteins encoded by plasmids of the pT26‐2 family led to the classification of the 902 putative proteins into 356 families (Supporting Information Datafile S1 and Fig. S4). A majority of them (696 proteins) belong to the variable regions (Supporting Information Datafile S1). For instance, among the 356 identified proteins families, 309 correspond to proteins present in less than three plasmids of the pT26‐2 family and 244 correspond to singletons. Most of these putative proteins, especially singletons, have small size, suggesting that their putative genes could be false ORFans, putative protogenes or new genes that recently originated de novo from proto‐genes (Supporting Information Fig. S4). The few large genes present in the variable regions encode integrases (FamAll_0015, 24 and 70 in Supporting Information Datafile S1) and putative replication proteins (FamAll_0034 and 103 in Supporting Information Datafile S1).

### *Identification of DNA replication modules*

#### *Putative DNA replication proteins*

We identified three different types of genes encoding putative replication (Rep) proteins among plasmids of the pT26‐2 family, one corresponding to hypothetical proteins specific for plasmids of this family and two corresponding to various proteins of the minichromosome maintenance (MCM) family that includes both archaeal and eukaryotic replicative helicases.

The first group is only present in four plasmids from Thermococcales and corresponds to the putative replication protein t26‐22p previously detected in the prototype plasmid pT26‐2 (Soler *et al*., 2010). These proteins are composed of a large central P‐loop NTPase domain framed by two short N‐ and C‐terminal domains that have no detectable sequence similarities with other proteins in databases. A homologous central P‐loop NTPase domain is present in primase/helicase Rep proteins encoded by pTIK4 and pORA1 plasmids of *Sulfolobus neozealandicus* (Greve *et al*., 2005). For these two *Sulfolobus* Rep proteins, the central domain is associated in the N‐terminus with a PrimPol domain that exhibits primase and polymerase activity (Lipps *et al*., 2004; Supporting Information Fig. S5). Although we could not detect the classical signature of PrimPol primases in the four Thermococcales proteins, the presence of the central P‐loop NTPase domain suggests that t26‐22p is a novel type of Rep protein with helicase activities fused to an additional domain of unknown function, possibly corresponding to a novel type of primase. The presence of the Rep protein t26‐22p in only four Thermococcales plasmids of the pT26‐2 family (Fig. 3 and Table 3) makes it an unlikely ancestral replication module.

**Table 3:** Conserved features among the pT26‐2 family.

Name	Relative core size in BDBH	Replication	Ori rep	Integrase type	Target tRNA
PspNA2_IP1	7	MCM	1	Type I	tRNA‐Val
TbaCH5_IP1	3	–	Not found	Type I	tRNA‐Val
TceDSMA7994_IP1	6	T26‐22p like	1	Type I	tRNA‐Thr
PchCG74_IP1	7	–	1	Type I	tRNA‐Gly
PHV1	8	–	1	Type I	tRNA‐Ala
PyaCH1_IP16	9	–	1	Type I	tRNA‐Gly
TbaCH5_IP2	9	MCM	1	Type I	tRNA‐Tyr
PabGE2_IP2	7	T26‐22p like	1	Type I	tRNA‐Ala
PkuNCB100_IP1	7	–	1	Type I	tRNA‐Ala
TliDSM5473_IP1	8	–	Not found	Type I	tRNA‐Gly
pT26‐2	8	T26‐22p like	1	Type I	tRNA‐Arg
TguDSM11113_IP1	9	–	1	Type I	tRNA‐Arg
TGV1	9	–	1	Type I	tRNA‐Arg
TKV3	8	T26‐22p like	1	Type I	tRNA‐Arg
TKV2	8	–	1	Type I	tRNA‐Glu
TspJCM11816_IP1	7	–	1	Type I	tRNA‐Arg
MmaOS7_IP1	9	MCM like	1	Type II	tRNA‐Ser
MmaKA1_IP1	8	MCM like	2	Type II	tRNA‐Ser
MmaX1_IP1	9	MCM	Not found	Type II	–
MMC7V1	7	MCM	Not found	Type II	tRNA‐Ser
MmaS2_IP	8	MCM	1	Type II	tRNA‐Ser
MMC6V1	9	MCM	1	Type II	tRNA‐Ser
MMC7V2	8	MCM like	1	Type II	tRNA‐Ser
MmaC5_IP1	2	–	Not found	–	–
MVV1	7	–	2	Type II	tRNA‐Leu
MthDSM2095_IP1	8	MCM like	1	Type II	tRNA‐Ser
pMEFER01	9	MCM	1	Type II	–
MspFS406‐22_IP1	8	–	1	Type II	tRNA‐Ser
MigKol5_IP1	8	–	1	Type II	tRNA‐Ser

The most represented Rep protein identified in plasmids of the pT26‐2 family corresponds to a MCM replicative 5′ to 3′ helicase which is found in both Thermococcales and Methanococcales elements (Figs. 3 and 4 and Table 3). These proteins are also encoded by MGEs from other families in Thermococcales, such as the plasmid pTN3 (Gaudin *et al*., 2014), the virus TPV1 (Gorlas *et al*., 2013) and the integrated plasmid TKV1 (Fukui *et al*., 2005) and by MGEs from other archaeal lineages (Krupovič *et al*., 2010; Krupovic *et al*., 2019). Moreover, one or several genes encoding MCM proteins are present in all archaeal genomes and correspond to chromosomal replicative helicases (Raymann *et al*., 2014).

We have performed two updated phylogenetic analyses of MCM proteins, one including all MCM encoded by archaea and their MGEs and the other focusing on MCM encoded by MGEs from Thermococcales and Methanococcales.

It has been previously shown that viral/plasmidic archaeal MCMs were recruited several times independently from their hosts during archaeal evolution (Krupovič *et al*., 2010). Confirming this result, MCM encoded by MGEs from Thermococcales and Methanococcales branch as sister groups of MCMs from their respective cellular hosts in our phylogenetic tree (Supporting Information Figs. S6–S8). In addition, as previously observed in Methanococcales (Krupovič *et al*., 2010), the MCM history is complex with the presence of both cellular and MGE paralogues as well as several cases of clear‐cut HGT (Supporting Information Fig. S6). In Thermococcales, MCMs encoded by MGEs (including those of the pT26‐2 family) form a monophyletic group but the two MCMs encoded by elements of the pT26‐2 family (PspNA2_IP1 and TbaCH5_IP2) do not cluster together (Supporting Information Fig. S7). Our phylogenetic analysis indicates that transfer of the *mcm* gene between MGE and their hosts took place early in Thermococcales evolution, before the separation between *Thermococcus* and *Pyrococcus* genera.

In Methanococcales, our phylogeny confirms the duplication of the MCM gene before the last Methanococcales ancestor (Krupovič *et al*., 2010; Walters and Chong, 2010; Supporting Information Fig. S8). In contrast to Thermococcales, MCMs encoded by Methanococcales MGEs are mixed phylogenetically with the two chromosomal MCM paralogues (MCM1 and MCM2) and cluster in three different groups (Supporting Information Fig. S8). This indicates that exchange of *mcm* genes has occurred more frequently in Methanococcales than in Thermococcales. Interestingly, the basal position of MCM proteins encoded by some plasmids of the pT26‐2 family in both Thermococcales and Methanococcales suggests that a host MCM could correspond to the ancestral replication protein of this plasmid family.

Finally, four plasmids of the pT26‐2 family present in Methanococcales encode a new family of distantly related MCM‐like proteins (Fig. 4 and Table 3), previously identified in bacteria (Mir‐Sanchis *et al*., 2016) and Thaumarchaea (Krupovic *et al*., 2019) MGEs. Notably, the bacterial MGEs also encode a serine recombinase downstream of the MCM‐like replication gene, responsible for the integration activity. The proximity of the replication and integration modules was proposed to facilitate replication after excision, enhancing transfer efficiency (Mir‐Sanchis *et al*., 2016). A similar gene layout is observed in the four plasmids of the pT26‐2 family, where the MCM‐like gene is located next to a tyrosine recombinase gene. Such organization is not observed in plasmids of the pT26‐2 family encoding the classical MCM replication protein. As the presence of this replication protein is restricted to four plasmids, it probably does not correspond to the ancestral replication protein, suggesting a more recent acquisition in Methanococcaceae.

Overall, we identified a putative Rep proteins in 15 out of 29 plasmids of the pT26‐2 family, including the three free plasmids (Table 3). The absence of a putative Rep protein in a particular integrated plasmid can be due to the presence of a novel type of Rep protein or due to the fact that these plasmids have lost the ability to replicate autonomously. The second hypothesis is supported for several integrated plasmids by the fact that they do not encode large proteins of unknown function in their variable regions, and/or have no detectable replication origins (see below). Taken together, these analyses reveal a complex evolutionary scenario for the replication module of plasmids of the pT26‐2 family, with several replacements that could correspond to new gene acquisitions. This high frequency of replication module replacement could partially compensate the observed tendency of these plasmids to lose the replication protein following integration.

#### *Origins of replication*

A putative replication origin (ori) was predicted by cumulative GC skew analysis for the prototype plasmid pT26‐2 between the *t26‐20p* and *t26‐21p* genes (Soler *et al*., 2010). To predict putative *ori* for each plasmids of the pT26‐2 family, we used two complementary methods (GC‐skew analysis and looked for repeat‐rich regions, see Methods section for more information). Together, these methods allowed us to identity a putative *ori* for 24 of the 29 plasmids of the pT26‐2 family (Supporting Information Table S1 and Fig. S9). Around half of these *ori* regions were identified by both methods independently, although for two elements, the two methods gave two different *ori* locations (Supporting Information Table S1 and Fig. S9). The majority of the remaining putative *ori* were predicted by GC‐skew analysis, and for five elements, we could not detect any putative *ori* with either method (Supporting Information Table S1 and Fig. S9).

Most predicted *ori* are located in intergenic regions or in regions containing multiple small open reading frames, which are potentially non‐coding (Figs. 3 and 4). Comparative analysis of the identified putative *ori* does not reveal any conserved consensus sequence. Given the low conservation of the non‐core region, it is difficult to say whether *ori* location is conserved between the different elements – even for the closely related elements in *M*. *maripaludis*, the putative ori location is variable (Figs. 3 and 4).

Overall, both the location and sequence of putative *ori* in plasmids of the pT26‐2 family seem extremely variable. However, we still observed a linkage between the putative *ori* and the replication protein; in most cases (12 of 15), the *ori* was located nearby the gene encoding one of the three types of putative Rep proteins.

### *Two integration strategies for the plasmids of the pT26‐2 family*

All plasmids of the pT26‐2 family encode an integrase of the tyrosine recombinase superfamily. So far, several families of tyrosine recombinases encoded by viruses and plasmids have been described in archaea (She *et al*., 2004; Erauso *et al*., 2006; Cossu *et al*., 2017; Wang *et al*., 2018). They are divided in two major types based on the strategy of integration (She *et al*., 2004): for type I integrases, recombination of the circular element with the host chromosome leads to division of the integrase gene into two fragments, a longer Int(C) fragment and a shorter Int(N) fragment; in contrast, the type II integrases maintain an intact integrase‐encoding gene after recombination. Both type I and type II integrases were previously detected in plasmids from the pT26‐2 family in Thermococcales and Methanococcales respectively (Soler *et al*., 2010). In order to determine if this observation was still valid for our extended dataset, we carried out a clustering analysis based on pairwise protein similarity. Beside the integrases that we detected in plasmids of the pT26‐2 family, we included in our dataset known archaeal integrases from different families and putative integrases that show sequence similarity to the pT26‐2 integrase (Int^pT26‐2^) but encoded by plasmids and/or viruses from different MGE families. Clustering analysis was performed using SiLiX with a minimum threshold of 25% identity over 40% of the protein (Miele *et al*., 2011). The result confirmed that the pT26‐2‐encoded integrases in Thermococcales and in Methanococcales correspond to two different types, as they are not connected to each other (Supporting Information Fig. S10). Rather, the integrases of plasmids present in Thermococcales were connected to the SSV‐integrase family (type I), whereas the integrases of plasmids present in Methanococcales were connected to XerC proteins, and less stringently, to the SNJ2 and pNOB8 integrases (type II).

Phylogenetic analysis confirmed the results of the network analysis, with Thermococcales integrases of the pT26‐2 family forming a sister group to pTN3 integrases and Methanococcales integrases of the pT26‐2 family forming a sister group to SNJ2 integrases (Supporting Information Fig. S11). The phylogenetic analysis also highlights several cases of integrase exchanges between different type of mobile elements (Supporting Information Fig. S11). For example, the integrases of *M*. *maripaludis* MMC7V2 and other *M*. *maripaludis* plasmids of the pT26‐2 family are closely related to the integrase of a *Methanococcus vannielli* provirus, suggesting an integrase exchange between the pT26‐2‐related plasmids and the virus.

For all site‐specific integrases, the recombination sites (att sites) exhibit identical sequences on the plasmid (attP) and the host chromosome (attB). Close examination of the att sites of the pT26‐2 family plasmids and of their integrase‐encoding genes confirmed the two different recombination strategies used by these plasmids. In Thermococcales, the attP site is located inside the coding sequence of the integrase gene, as expected for type I integrases, whereas in Methanococcales, the att sites are often located close to the integrase gene as expected for type II integrases. However, in some cases, the att site of type II integrases was located further away from the *int* gene. For instance, in *M*. *maripaludis* elements, the *int* gene is positioned in the middle of the integrated element.

**Figure 6:**
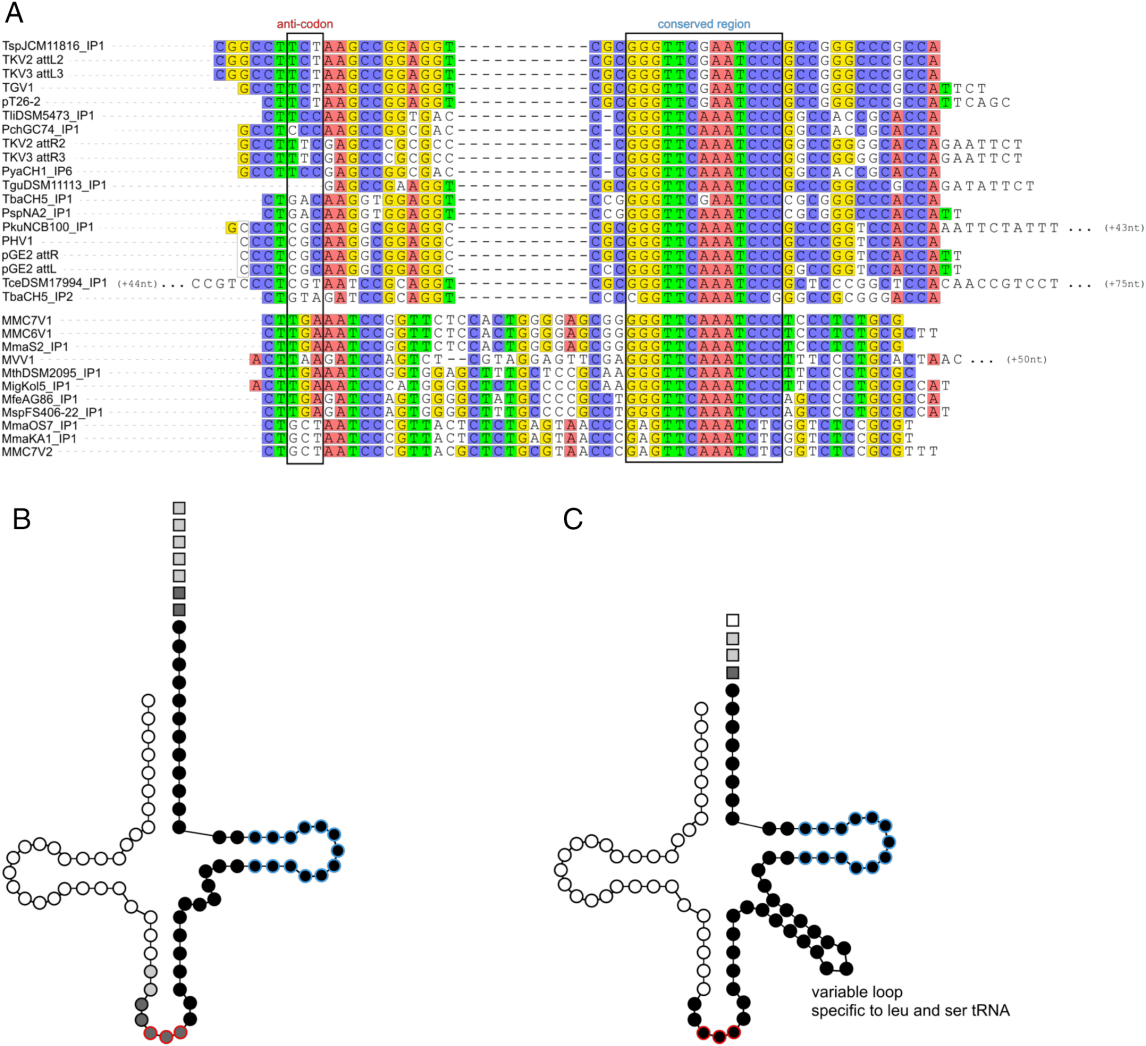
att site variability. A. The att sites correspond to the 3′ terminus of the tRNA genes. On the alignment, the anti‐codon is framed. The consensus sequences among Thermococcales and among Methanococcales att sites are highlighted in colour. Long sequences were only partially presented. B and C. The att sites are displayed on the structure of the targeted tRNA for Thermococcales and Methanococcales respectively. Circles represent tRNA nucleotides. Red circles correspond to the anti‐codon. Squares represent att sites nucleotides downstream of the tRNA gene. Black nucleotides are present in all att sites, darker grey in more than 77% and lighter grey in more than 33%. [Color figure can be viewed at http://wileyonlinelibrary.com]

For both types I and II integrases, the attB sites are usually located at the 5′ or 3′ regions of tRNA genes (Faraco *et al*., 1989; She *et al*., 2004). The alignment of the different att sites from Thermococcales and Methanococales plasmids of the pT26‐2 family showed that an imperfect palindromic sequence is conserved among the different att sites (Fig. 6A). This sequence corresponds to the two T‐stems of the T‐arm in the 3′ region of the tRNA (Fig. 6B and C). All attB sites of pT26‐2 family plasmids overlap with the 3′ end of a tRNA gene (Fig. 6 and Table 3), most often including the anti‐codon sequence. As observed for other integrase families, the tRNA genes are not disrupted by the integration event (Schleper *et al*., 1992).

The att sites of Thermococcales plasmids average 58 nt in length, similar to that previously observed with SSV1 and pTN3 integrases (Schleper *et al*., 1992; Cossu *et al*., 2017). The Thermococcales attB sites are present in a wide variety of tRNA genes, including Arg, Thr, Gly, Val, Glu, Tyr and Ala tRNA genes (Supporting Information Fig. S12a; Table 3). In contrast, all attB sites in Methanococcales correspond to Ser tRNA genes, with the single exception of a Leu tRNA gene for MVV1. The att sites are longer in Methanococcales plasmids, accounting for the presence of a variable loop in the tRNA‐Leu and tRNA‐Ser recognized by their integrases (Fig. 6). Our results indicate that Thermococcales pT26‐2 integrases present a high diversity of att sites, with no clear preferential target tRNA. In contrast, the att sites of Methanococcales pT26‐2 integrases are presently limited to tRNAs containing the additional variable loop (Ser‐tRNA and Leu‐tRNA).

## Discussion

The family of archaeal plasmids epitomized by the element pT26‐2 was first described following the isolation of *Thermococcus* sp. 26‐2 (Soler *et al*., 2010). Here, we expand our knowledge of this plasmid family by identifying new integrated plasmids in Thermococcales and Methanococcales genomes and describe the first free pT26‐2 family member present in a *Pyrococcus* strain, namely pGE2 from *Pyrococcus* sp. GE2.

All plasmids of this family are formed by the association of a variable region that often includes the Rep protein and a putative replication origin and a conserved region, the ‘core module’, rich in genes encoding several putative membrane proteins and an ATPase that could be involved in DNA transfer. The core module include seven genes that are conserved in all elements and can be used to define the pT26‐2 family. Phylogenetic analyses of these pT26‐2 core proteins reveal a well‐supported bipartition of Thermococcales and Methanococcales, suggesting that these plasmids have evolved independently in each taxonomic group after the separation of the two lineages and were never transferred between members of the two orders. This cannot be explained by their geographic distribution as Thermococcales and Methanococcales hosts of plasmids from the pT26‐2 are often present at the same location (Fig. 1).

The hypothesis of independent plasmid evolution in Thermococcales and Methanococcales is further supported by several observations. (i) All CRISPR spacers directed against plasmids present in strains of one order (Thermococcales or Methanococcales) are specific for plasmids detected in this order; (ii) phylogeny of the Rep proteins shared between pT26‐2 plasmids of these two orders (MCM) also show a clear‐cut separation between them, and non‐MCM Rep proteins are specific either for Thermococcales (t26‐22p‐like protein) or Methanococcales (the distantly related MCM‐like protein and PCNA) and (iii) Thermococcales and Methanococcales plasmids of the pT26‐2 family are characterized by different types of integrases (types I and II respectively) and different integration specificities.

**Figure 7:**
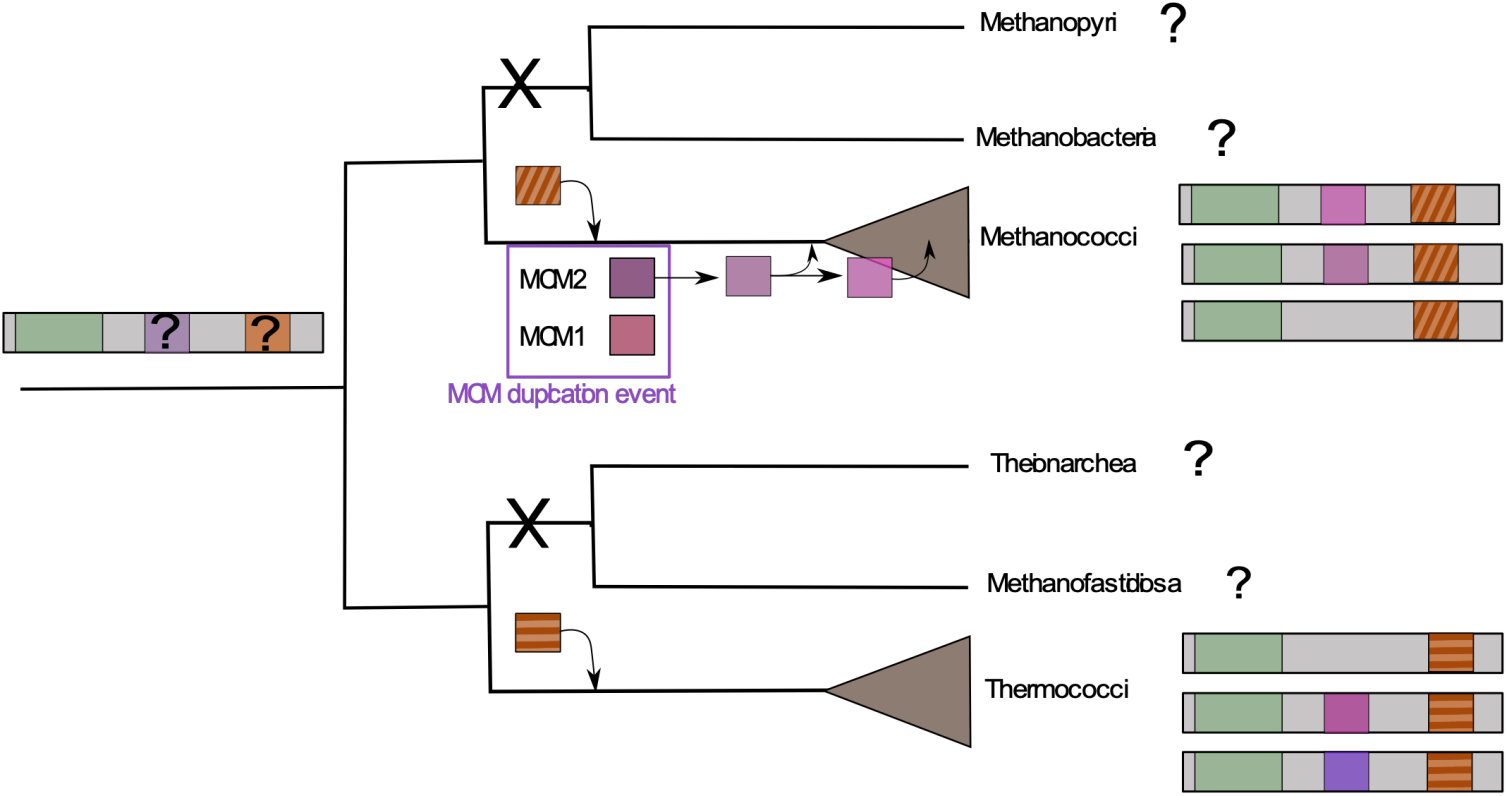
The evolutive model of the pT26‐2 family. In this schematic representation, the core module and the integrase module are indicated in green and orange respectively. The different replication modules are indicated with different shade of purple. [Color figure can be viewed at http://wileyonlinelibrary.com]

Two observations suggest that ancestral plasmids of the pT26‐2 family were already present in the last common ancestor of Thermococcales and in the last common ancestors of Methanococcales. Firstly, the plasmids of the pT26‐2 family and CRISPR spacers which target them are widespread in both orders, and secondly, our phylogenetic analysis indicates that the core proteins have co‐evolved with their hosts without inter‐order transfers. Taken together our results lead us to propose two evolutionary models for the pT26‐2 family (Fig. 7). The first model, which we favour, proposes that the core module was already encoded by an ancestral plasmid (ancestral‐pT26‐2) present in the last common ancestor of Methanocococales and Thermococcales. This ancestral pT26‐2 plasmid probably contained a replication module, potentially an MCM helicase. We observed that during the evolution in the Methanococcales, the pT26‐2 element has replaced its replication protein with the host chromosomal MCM2 and with another kind of MCM‐like replication protein from an unknown source. In some plasmids of Thermococcales, this replication protein has been replaced by a t26‐22p‐like protein. For the integration module, we can formulate two evolutionary hypotheses: (i) an integrase could have been present in the ancestral pT26‐2 and then replaced in the ancestral Methanococcales pT26‐2 and/or the ancestral Thermococcales pT26‐2 or (ii) the integration module could have been absent in the ancestral‐pT26‐2 and then acquired twice independently in the ancestral Methanococcales pT26‐2 and ancestral Thermococcales pT26‐2. In this first model, the independent loss of plasmids of the pT26‐2 family in Methanobacteriales and Methanopyrales could be explained by the appearance of a unique cell wall consisting of pseudomurein in Methanobacteriales and Methanopyrales (Steenbakkers *et al*., 2006; Visweswaran *et al*., 2010). Our second model posits that two ancestral elements sharing the same core genes were introduced independently in ancestors of Thermococcales and of Methanococcales.

A clear case of horizontal plasmid transfer between *Thermococcus* and *Methanocaldococcus* was previously described for the pTN2 family, based on comparative genomics (Krupovic *et al*., 2013). In contrast, our work suggests that the highly conserved region of plasmids of the pT26‐2 family has never been transferred between Methanococcales and Thermococcales. Despite the abundance of plasmids of the pT26‐2 family within these taxonomic groups, their mobility appears to be prohibited at an inter‐order scale. We could identify few cases of HGT between different members of the Thermococcales. Similar to the core module, the integration and replication modules of plasmids of the pT26‐2 family have also evolved within the order boundaries. Nevertheless, they exhibit a more complex evolutionary history with many apparent exchanges with plasmids and/or viruses from other families of the same order. It was originally reported that DNA exchange was preferentially observed within ‘DNA vehicles’ of the same type (chromosome, plasmid or virus; Halary *et al*., 2009). However, in the last decade, data suggesting a strong evolutionary connection between plasmids and viruses have accumulated. MGEs have a modular organization, and each module can follow its own evolutionary history by recombinational exchange with other mobile elements and/or their host genome (Guérillot *et al*., 2013; Iranzo *et al*., 2016). Here, our analysis shows that the integration and replication modules of plasmids of the pT26‐2 family have been exchanged with the host chromosome and with other integrated elements, some of which have been identified as viruses sharing the same host.

We did not detect any plasmids of the pT26‐2 family in any other archaeal phylum or in Bacteria. In particular, they are not present in MAGs of Theinoarchaea and Methanofastidiosa, which are closely related to Thermococcales in most recent phylogenetic analyses (Adam *et al*., 2017). However, only a limited number of partial MAGs are presently available for these two new candidate orders (Nobu *et al*., 2016; Lazar *et al*., 2017) and their future exploration might reveal new MGEs related to those of Thermococcales. Moreover, as our knowledge on the diversity of archaea and their mobilome is rapidly increasing, one can expect that plasmids of the pT26‐2 family may be finally identified in other archaeal lineages.

We noticed a tendency of integrated plasmids of the pT26‐2 family to lose their replication ability upon integration in both host orders, although more strongly so in Thermococcales. The latter observation could be linked to the integration mechanism employed by Thermococcales which appears to be suicidal. The excision and recircularization of Themococcales plasmid of the pT26‐2 family after integration seems more difficult as the integrase gene is split during the integration process. Notably, the core module is still strictly conserved in several integrated plasmids that have lost their Rep protein and/or their putative replication origin. This could reflect some selective advantage that favours the conservation of this core module. In addition, the conservation of the all core genes, even with the addition of the new pT26‐2 family members may indicate that the core module is maintained by natural selection as a single unit. All these observations support the idea that this core module confers a selective advantage to the plasmid and potentially to the hosts of the integrated plasmids.

In 2013, genetic studies of mutants lacking each of the four plasmids integrated in the genome of *T*. *kodakarensis* showed that deletion of TKV2 and TKV3 (both from the pT26‐2 family) negatively effects growth (Tagashira *et al*., 2013). This suggests that the presence of these integrated plasmids stimulate cell growth, at least under laboratory conditions. One possibility is that the core module could still be used as a gene transfer agent, as a new kind of secretion system, or as a kind of interaction system between different cells. The core module of the pT26‐2 family indeed encodes several predicted transmembrane proteins that may be involved in the formation of a unknown DNA secretion system.

The genetic organization of pT26‐2 is reminiscent of that of the infectious plasmid pR1SE recently isolated from vesicles of the halophilic archaeon *Halorubrum lacusprofundi* R1SE, albeit without any direct sequence conservation (Erdmann *et al*., 2017). It was experimentally shown that pR1SE can promote its own transfer between cells via extracellular vesicles which contain multiple transmembrane proteins encoded by the plasmid itself (Erdmann *et al*., 2017). Other plasmids in Thermococcales species, such as the pTN3, have been shown to use membrane vesicles to transfer horizontally (Gaudin *et al*., 2014), and two species containing integrated plasmids of the pT26‐2 family, *Thermococcus gammatolerans* and *Pyrococcus horikoshii*, were shown to produce membrane vesicles containing cellular DNA (Soler *et al*., 2008). Moreover, the DNA within the vesicles of *T*. *gammatolerans* is resistant to DNase treatment and thermodenaturation as compared with naked DNA (Soler *et al*., 2008). It is thus tempting to suggest that plasmids of the pT26‐2 family could also use extracellular vesicles for dissemination. Vesicle protection could facilitate passive transport under the harsh environmental conditions that prevail in Thermococcales habitats. This protection may also help to explain the global geographic distribution of pT26‐2 family, similar to marine viruses where capsid proteins protect DNA during transport along oceanic currents (Brum *et al*., 2015). Alternatively, plasmids of the pT26‐2 family could encode a novel type of DNA transfer mechanism allowing for direct transfer of plasmid DNA through cell membranes via cell to cell contact. In the future, it will be important to revive integrated plasmids of the pT26‐2 family or to identify stable free plasmids of this family to test these hypotheses.

## Methods

### *Isolation, sequencing and detection of integrated copies of pGE2 in GE2*

Plasmid pGE2 was isolated from a 50 ml of *P*. *abyssi* strain *Pyrococcus* GE2 culture in late exponential growth phase, using a modified alkaline‐lysis method as previously described (Erauso *et al*., 1996). A shotgun plasmid library of clones of pGE2 was constructed in pUC18 vector and sequenced from both ends as described previously (Gonnet *et al*., 2011). The complete plasmid sequence was deposited to the GenBank under the following accession numbers: MN477947.

Around 10 years ago, the pGE2 copy number was estimated using a real‐time quantitative polymerase chain reaction (PCR)‐based method (Lee *et al*., 2006; Providenti *et al*., 2006). Two set of primers were tested for pGE2‐specific primers targeting respectively the CDS7 (UVRD Helicase) and the CDS29 (putative Rep) and one pair, Arc344F‐Uni516R, targeting the 16S rRNA gene of GE2 (sequences of the primers are given in the Supporting Information Table S2). In primary cultures of strain GE2, pGE2 coexisted both as a free and an integrated copy. The free plasmid was present in up to ~40 copies per chromosome but it disappeared during subculturing. The average copy number of pGE2 at that time was essentially the same using either CDS7 or CDS29 primer pair.

The pGE2 integration site in a tRNA gene was determined using an inverse PCR strategy, and confirmed by the whole‐genome sequencing of the *P*. *abyssi* strain GE2 in the framework of a large project on comparative genomics of Thermococcales. Today the free plasmid could not be retrieved from the assembly and by the read mapping analysis. In addition, the integrated copy of the pGE2 plasmid was found to be slightly smaller (21,837 bp) than the free form established several years ago. The difference corresponds to the presence of an insertion sequence (IS) of 1,825 bp containing two genes, encoding a resolvase (cds 1; 163 amino acids) and a transposase (cds 2; 429 amino acids) respectively. This IS element belongs to the IS family IS*200*/IS*605* (Chandler and Mahillon, 2002) and is identical to the element found in *P*. *abyssi* GE5^T^ (PAB2076/PAB2077) and was also detected in the *P*. *abyssi* GE2 genome, suggesting that the copy found in the episomal form of pGE2, originated from the host chromosome.

### *pT26‐2 family update*

In order to identify new members of the pT26‐2 family (Soler *et al*., 2010), each of the seven previously identified core genes (t26‐5p, 6p, 7p, 11p, 13p, 14p and 15p) were used as query for homology search in complete archaeal genomes, using SynTax (Oberto, 2013), a web server linking protein conservation and synteny. In addition, we also screened by BLASTP search the NCBI non‐redundant protein database to access to plasmids and non‐complete genomes. Our strategy allows us to identify remnant element or would allow us to identify atypical related MGE (where some protein first though as the core could have been associated to other type of module). All DNA regions, even if they encode few putative core genes, were analysed. For each region, we determined their extremities by the identification of direct repeat sequences (att sites) resulting from the recombination reaction.

### *Limits detection and integrase extraction*

In Thermococcales, integrase genes were identified by homology with the integrase of the plasmid pT26‐2 (Int^pT26‐2^). Genomes were searched by tblastn using as query the N‐ter or C‐ter region of Int^pT26‐2^ and the subsequently identified integrases. Attachment sites (att) were identified as identical region on the border of integrase N‐ter and C‐ter coding regions. Up to three mismatches were accepted in the middle of the att site to take into account sequence degenerescence after integration. N‐ter coding sequences were defined from a start codon (ATG or GTG) to the last non att codon. C‐ter coding sequences were defined from the first att codon to a stop codon. Complete integrase genes were reconstructed by adjoining N‐ter and C‐ter coding regions. The N‐ter and C‐ter region did not have matching open reading frames only for TKV2 and TKV3.

In Methanococcales, no Int^pT26‐2^ homologues were identified by tblastn. Att sites were identified as identical sequences in proximity to the detected core genes. Up to three mismatches were accepted in the middle of the att site to take into account sequence degenerescence after integration. Annotated integrase genes were located in between the att sites. Additional integrases were searched by tblastn with annotated ones as query.

### *Read mapping and control of the inversion in the* T. kodakarensis *genome*

In *T*. *kodakarensi*s, the att sites of the two plasmids of the pT26‐2 family, TKV2 and TKV3, are unexpectedly mixed. TKV2 attL2 is not identical to TKV2 attR2 but to TKV3 attL3. Similarly, TKV2 attR2 is identical to TKV3 attR3 (Supporting Information Fig. S2). This can be explained by an inversion between the two integrated plasmids (Supporting Information Fig. S2). As the inversion affecting the TKV2, TKV3 orientation observed in the *T*. *kodakarensis* could be the result of an assembly problem, we mapped reads obtain from our laboratory strain against the NCBI available genome, using Bowtie 2 (Langmead and Salzberg, 2012). After mapping, we observed a 500X coverage along the integrated elements and at both limits so this results confirmed that the observed inversion do not results from an assembly mistake. We compared the read mapping coverage over the TKV2 and TKV3 elements and at the limits of the integrated plasmids compared with that observed for the rest of the *T*. *kodakarensis* genome. We observed no read mapping defect at the limits of the integrated elements, confirming that this inversion does not result from an assembly problem.

The same approaches was made on the *P*. *abyssi GE2* genome and on the *Themococcus* 26‐2 genome in order to detect a higher proportion of read mapping in the region corresponding to integrated pT26‐2 plasmid, unfortunately no clear higher coverage could have been observed.

### *Host specificity determination*

The host specificity was determined by the presence of a CRISPRspacer against each pT26‐2 element in the archaeal genomes present in the CRISPRdb database (Grissa *et al*., 2007; http://crispr.i2bc.paris-saclay.fr/). At the time of the analysis, the database contained 232 complete archaeal genomes, including 27 and 15 genomes of Thermococcales and Methanococcales respectively at the time of the analysis. The nucleotide sequences of all identified plasmids of the pT26‐2 family were compared by blastn approach against the spacer‐sequences in the CRISPR database.

### *Orthologous protein identification*

For each plasmid of the pT26‐2 family, the encoded proteins were extracted. In order to identify orthologous proteins, we used RBH a common strategy used in comparative genomics. Basically, a RBH is found when the proteins encoded by two genes, each in a different MGE, find each other as the best scoring match. NCBI's blast is the software most usually used for the sequence comparisons necessary to finding RBHs. The protein sequence comparisons were performed using the NCBI's blast version 2.2.28+, every blast score was normalized to the alignment of query and hit proteins to themselves. Proteins showing normalized bidirectional blast >30% were considered orthologous as recommended by Lerat and colleagues (2003). Then, we tested the impact of the selection of each different plasmid of the pT26‐2 family as a pivot MGE on the core protein number size (protein present in 80% of the tested plasmids). The comparative analysis showed that the number of ‘core genes’ is affected by the pivot selection and varies from 7 to 9. If TbaCH5_IP1 is selected as a pivot, the number of core gene falls to three reflecting the remnant state of this integrated element (Table 3).

### *SiLiX network*

SiLiX (Miele *et al*., 2011) is a programme developed to cluster homologous proteins into families based on blastp results. All‐against‐all blastp analyses were performed on all encoded pT26‐2 integrases with the addition of related integrases found in Thermococcales and Methanococcales and of an integrase and a part of the dataset recently used for the analysis of the SNJ2 integrase family (Wang *et al*., 2018). The all‐against‐all integrases blastp results were grouped using the SiLiX package v1.2.8 (http://lbbe.univ-lyon1.fr/SiLiX; Miele *et al*., 2011). This approach for the clustering of homologous sequences is based on single transitive links with alignment coverage constraints. Several different criteria can be used separately or in combination to infer homology separately (percentage of identity, alignment score or E‐value and alignment coverage). For this integrase dataset, we used the additional thresholds of 25% and 60% for the identity percentage and the query coverage respectively. The network was visualized using igraph package from R (https://igraph.org/). In order to find densely connected communities in a graph via random walks, we used the cluster_walktrap function of the igraph package.

### *Synteny conservation*

Synteny conservation among plasmids of the pT26‐2 family was preliminary analysed using SynTax a web server linking protein conservation and synteny in complete archaeal genome. Then the synteny conservation among integrated and non‐integrated plasmid of the pT26‐2 family was confirmed using easyFig a Python application for the comparison of genomic loci based on side‐by‐side visualization of blast results (Sullivan *et al*., 2011).

### *Putative protein function*

As the blastp comparison is not sufficient to succeed to infer putative functions to the Core proteins. All Core proteins sequences were analysed with Phyre2 (Kelley *et al*., 2015). Phyre2 is a suite of tools available on the web to predict and analyse protein structure, this tool compare the given sequences to a Hidden Markov Model HMM database of known structures.

### *Replication origin prediction and module analysis*

Replication origins are usually AT rich regions of low stability that contain multiple direct and inverted repeated sequences (Sun *et al*., 2006; Krupovic *et al*., 2013). The replication origin was determined by two complementary methods (Supporting Information Table S1): (i) GC‐skews where the replication origin correspond to peaks and (ii) looked for repeat‐rich regions by dotplot analysis with Gepard (Supporting Information Fig. S9).

To determine the origin of the MCM proteins encoded by plasmids of the pT26‐2 family, we performed phylogenetic analyses using the core MCM helicases predicted by Raymann *et al*. (Raymann *et al*., 2014) and additional MCM helicases encoded by various MGEs identified in Thermococcales and Methanococcales genomes (Krupovič *et al*., 2010), that belong to pT26‐2 plasmids or not. In order to focus on the Thermococcales and Methanococcales MCM histories, we made two separated phylogenetic analyses using the Theionarchaea and the Methanofastidiosa or the Methanobacteriales as an outgroup respectively (Supporting Information Figs. S7 and S8).

### *Alignments and trimming and phylogenetic analysis*

Each alignment used for phylogenetic analyses was performed using MAFFT v7 with default settings (Katoh and Standley, 2013) and trimmed with BMGE (Criscuolo and Gribaldo, 2010) with a BLOSUM30 matrix and the ‐b 1 parameter.

For the maximum likelihood analysis, IQ‐TREE v1.6 (http://www.iqtree.org/) was used with the best model as suggested by the best model selection option (Wong *et al*., 2017). Branch robustness was estimated with the nonparametric bootstrap procedure (100 replicates) or with SH‐like approximate likelihood ratio test (Guindon *et al*., 2010) and the ultrafast bootstrap approximation (1,000 replicates) (Chernomor *et al*., 2017).

## Supporting information


**Appendix S1**: Supporting Information

**Appendix S2**: Supporting Information

**Appendix S3**: Supporting Information
