# Supplementary material for: The global distribution and evolutionary history of the pT26‐2 archaeal plasmid family
Source: Environ Microbiol. 2019 Oct 21;21(12):4685–705. doi: 10.1111/1462-2920.14800 (PMC6972569; doi:10.1111/1462-2920.14800)
Supplement: Supplementary file 1 — Appendix S1: Supporting Information [file EMI-21-4685-s001.pdf]

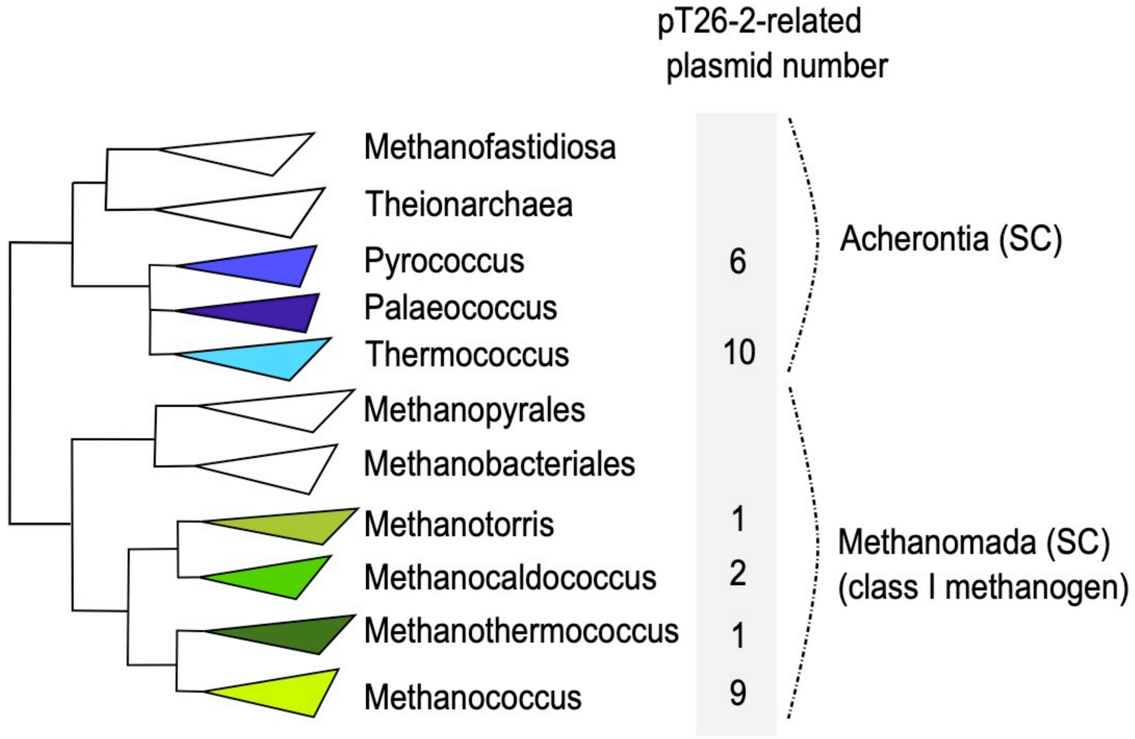

Fig. S1. Schematic representation of the phylogenetic relation between the Thermococcales, the Methanococcales and their closest archaeal relatives. In this schema the different genus of the Thermococcales and the Methanococcales were coloured in blue and green respectively

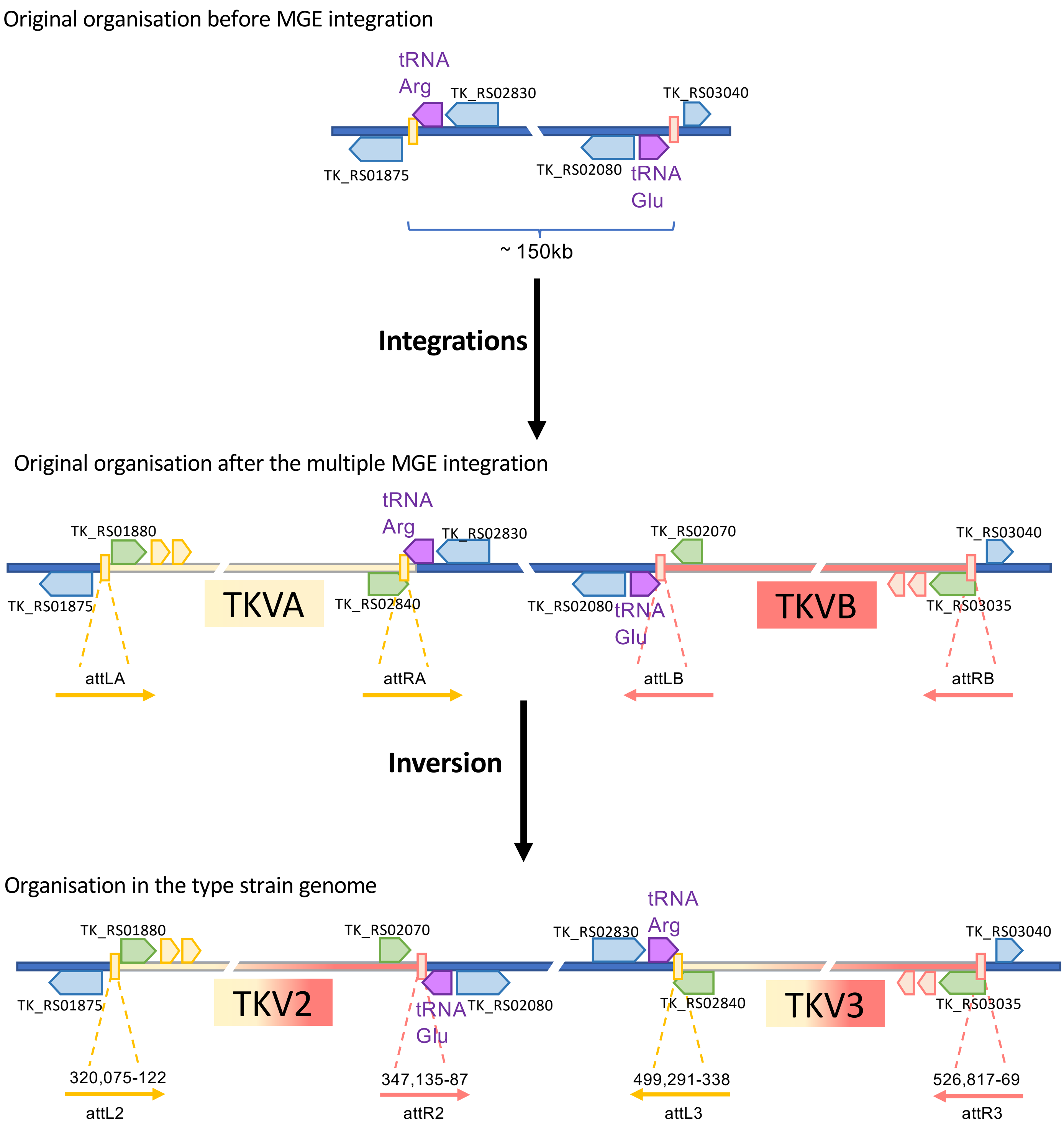

Fig. S2. TKV2 and TKV3 inactivation by large DNA inversion in *T. kodakarensis* genome.

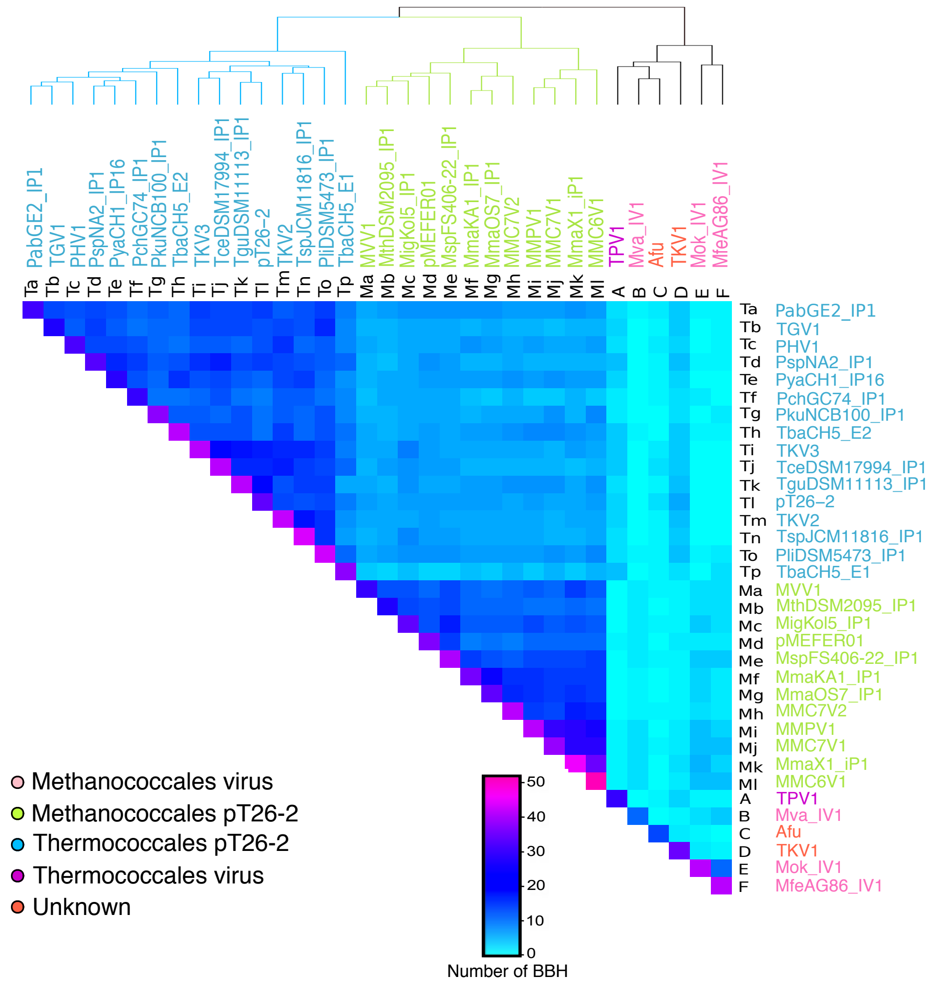

Fig. S3. Conservation of plasmids of the pT26-2and their connections with viruses by heatmap. Results of Bidirectional Best-Hit are represented as a heatmap. In the heatmap the number of conserved proteins is indicated in the scale. The heatmap analysis revealed two distinct groups, one containing the elements in Thermococcales and the other those of Methanococcales. The heatmap also show that some plasmid of the pT26-2 family shared genes with viruses.

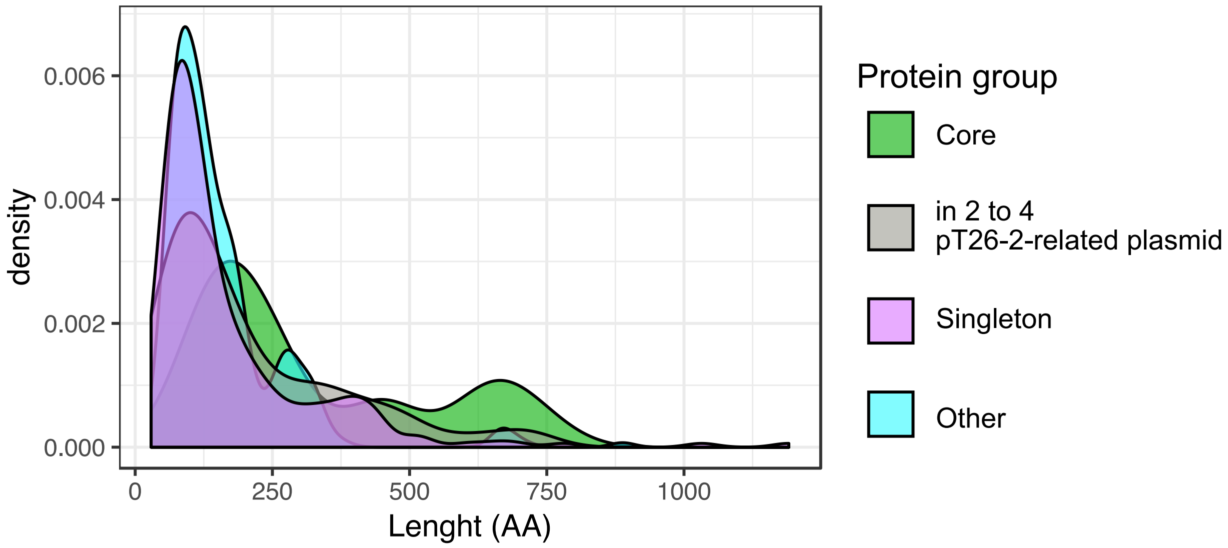

Fig. S4. Size protein encoded by plasmids of the pT26-2 family. The protein set were divided in 4 different groups. For each group the density of protein is indicated at the different protein size.

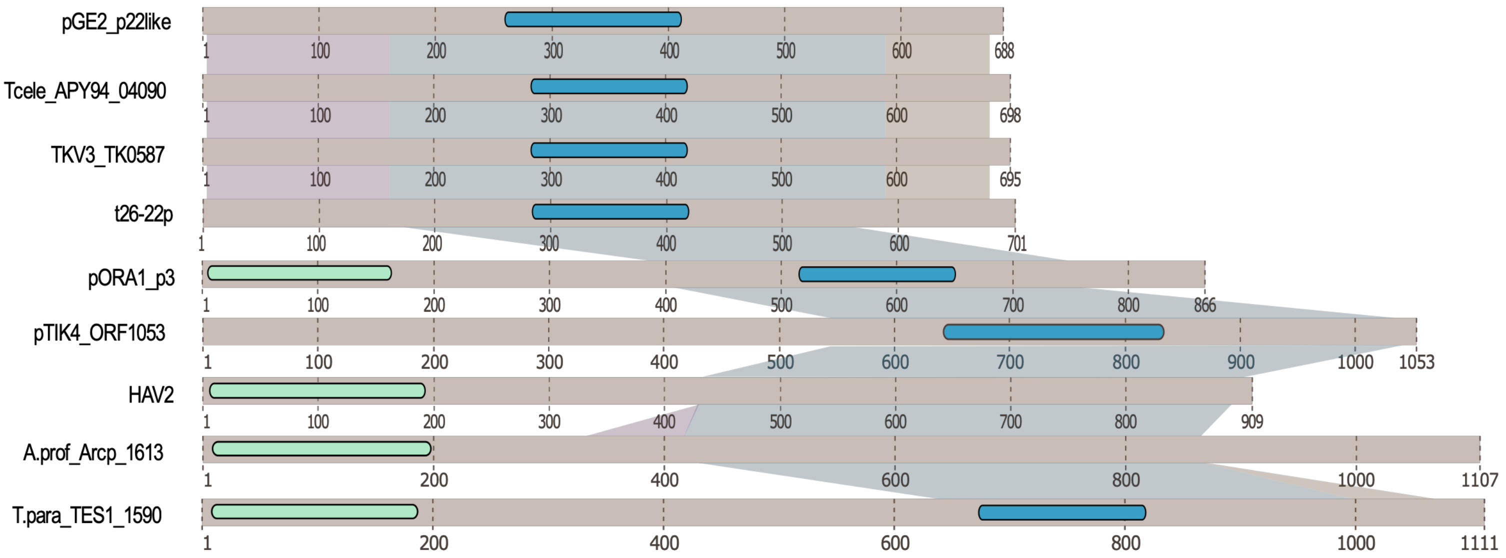

Fig. S5. Organisation of the pT26-2 putative replication protein and its comparison with other replication proteins. In this schematic representation the Primpol and the P-loop NTPase domain are indicated in green and blue respectively.

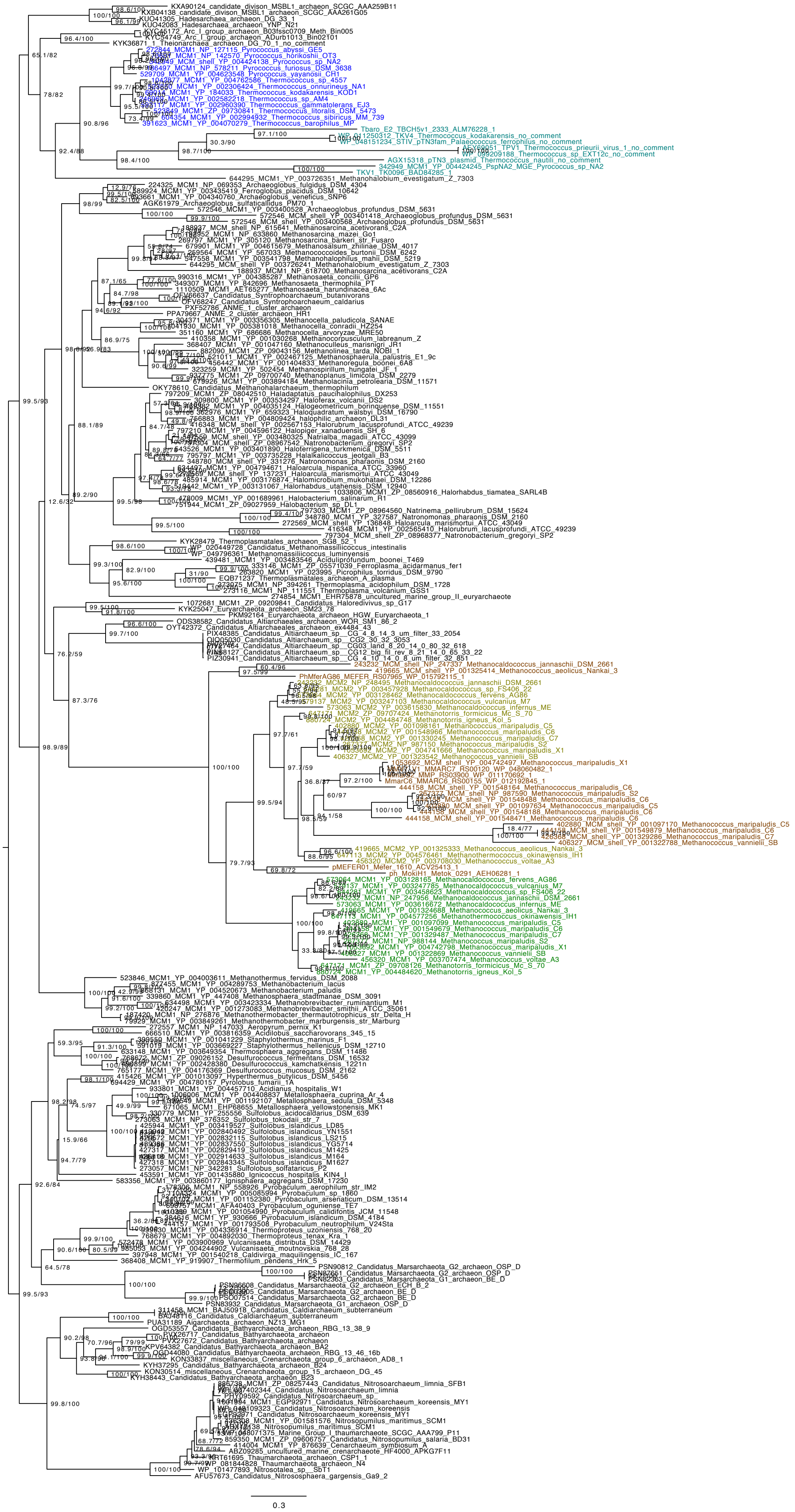

Fig. S6. MCM history within the Archaea. Maximum Likelihood phylogeny of the MCM protein within the Archaea. The Thermococcales chromosomic MCM are indicated in darkblue, the MCM encoded by Thermococcales MGEs are indicated in lightblue. The two Methanococcales chromosomic MCM are indicated with two different green. The Methanococcales MGEs encoded MCM are indicated in brown. The scale-bar represent the average number of substitutions per site. Values at nodes represent support calculated by aLRT and ultrafast bootstrap approximation (1,000 replicates).

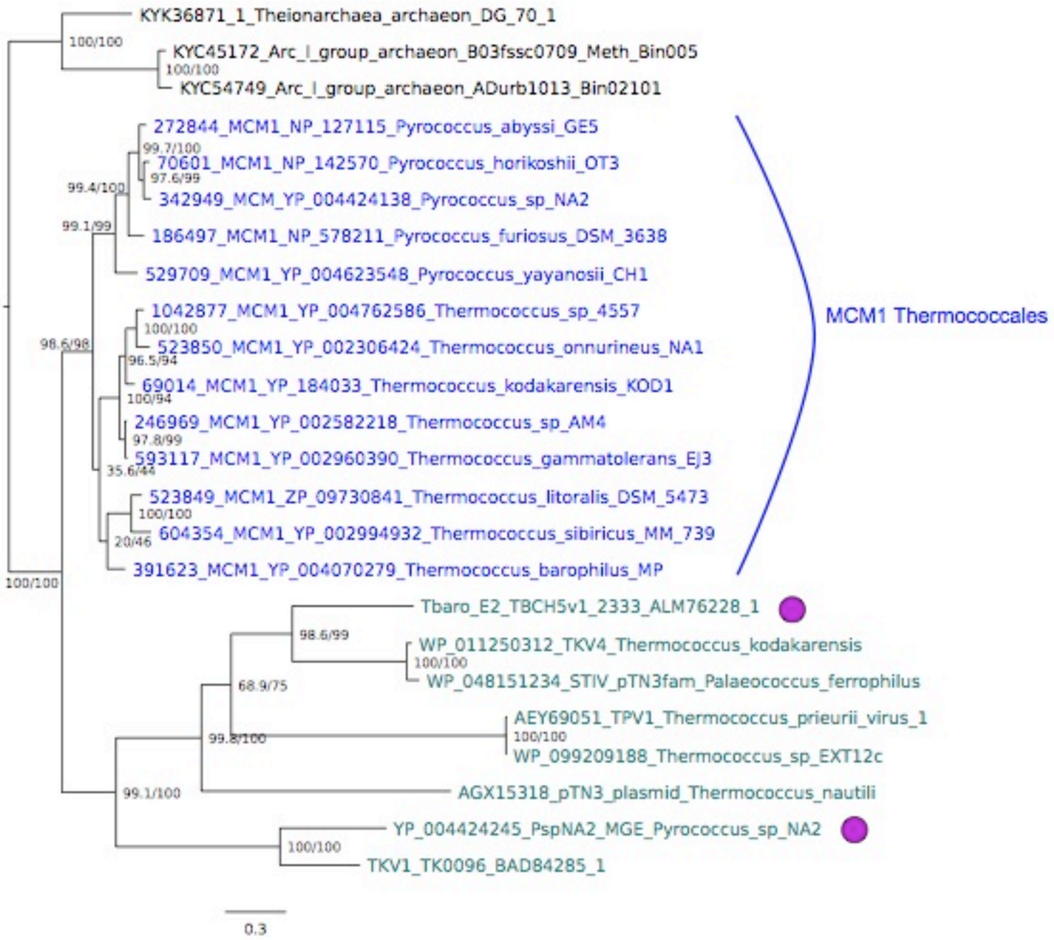

Fig. S7. MCM history within Thermococcales. Maximum Likelihood phylogeny of the MCM protein within Thermococcales using the *Theionarchaea* and the *Methanofastidiosa* as an out group. The Thermococcales chromosomic MCM are indicated in dark blue, the MCM encoded by MGEs are indicated in and the purple dots correspond to plasmids of the pT26-2 family. The scale-bars represent the average number of substitutions per site. Values at nodes represent support calculated by aLRT and ultrafast bootstrap approximation (1,000 replicates).

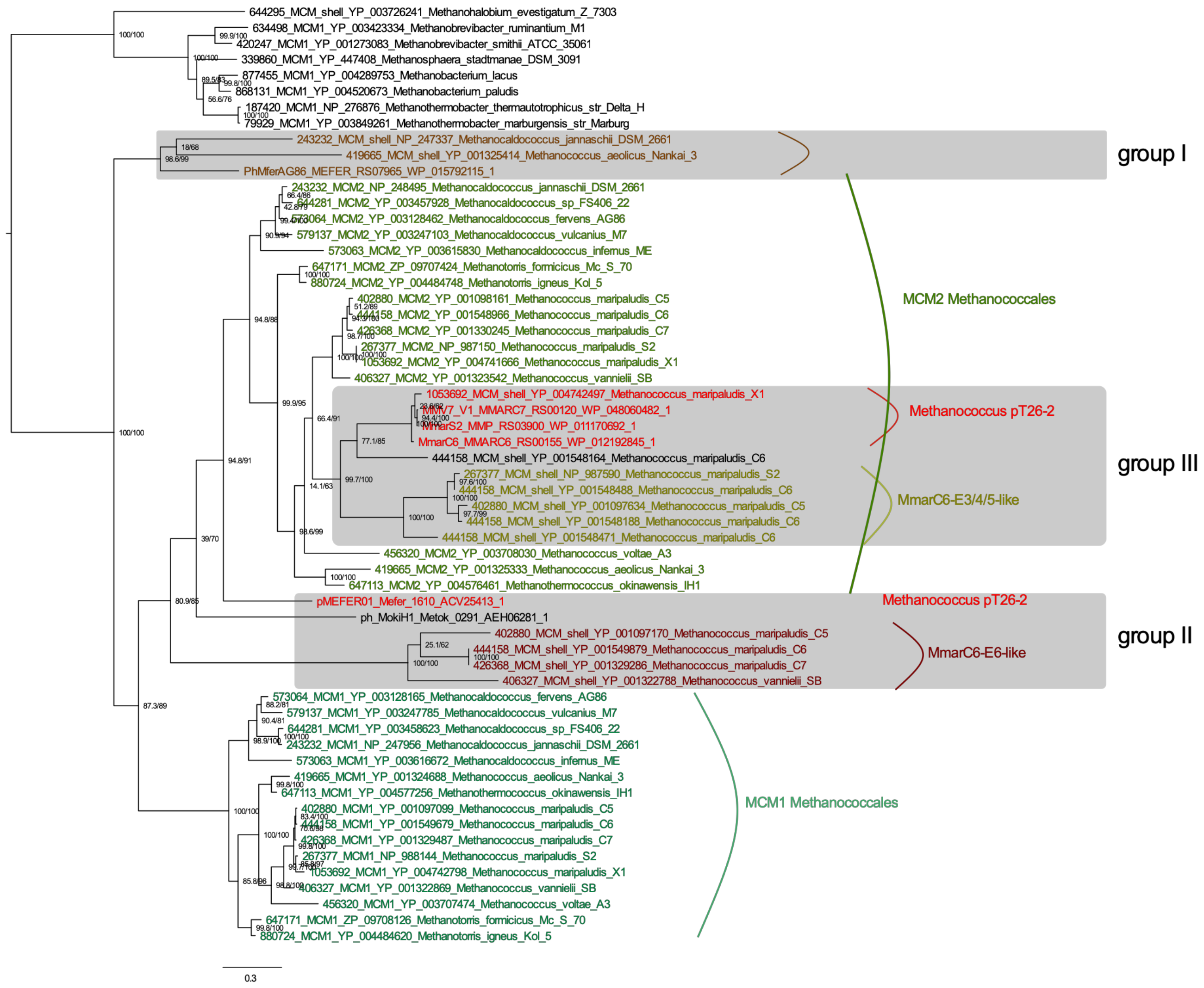

Fig. S8. MCM history within Methanococcales. Maximum Likelihood phylogeny of the MCM protein within Methanococcales using the Methanobacteriales as an out group. The two Methanococcales chromosomic MCM are indicated in dark green by the names MCM1 and MCM2. the MCM encoded by MGEs are indicated with several other colours and are all within three groups highlighted in grey. The MCM encoded by Methanococcales plasmids of the pT26-2 family are indicated in red. The scale-bars represent the average number of substitutions per site. Values at nodes represent support calculated by aLRT and ultrafast bootstrap approximation (1,000 replicates).

Fig. S9. Putative Replication origin prediction by GC-skew (left) and dotplot (right) in plasmid of the pT26-2 family. For integrated elements, the zero coordinate correspond to the first nucleotide of attL. Dotplots were drawn with Gepard.

pT26-2 plasmid

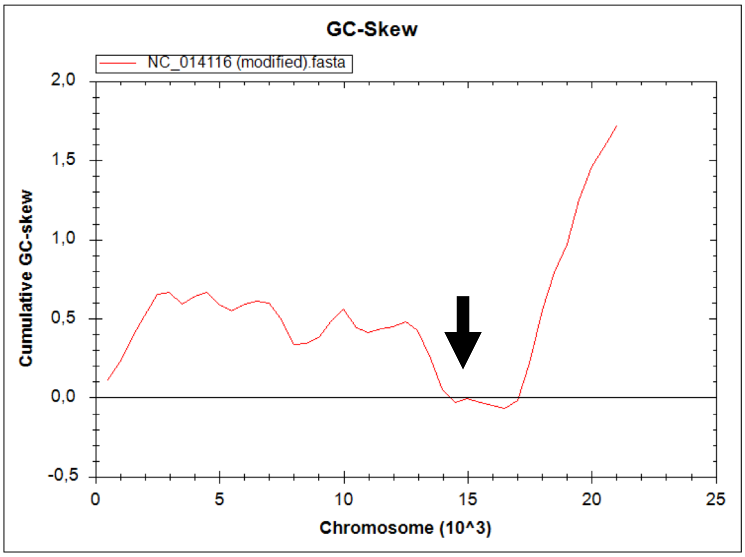

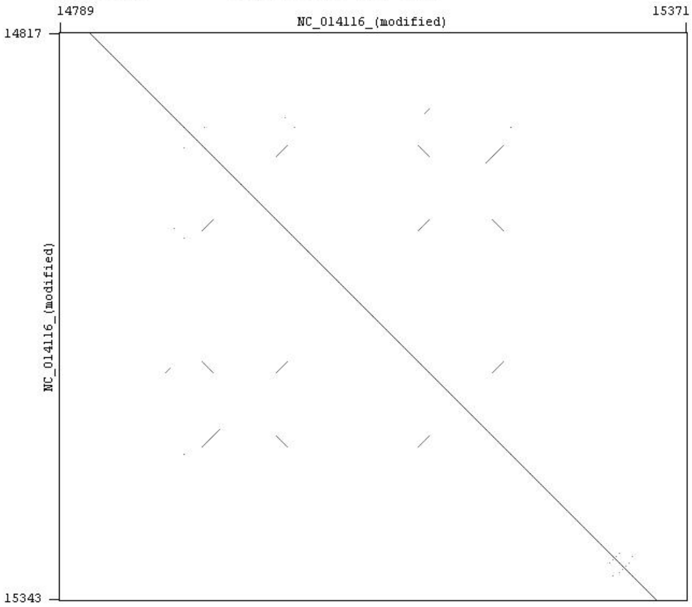

Thermococcus kodakarensis TKV1

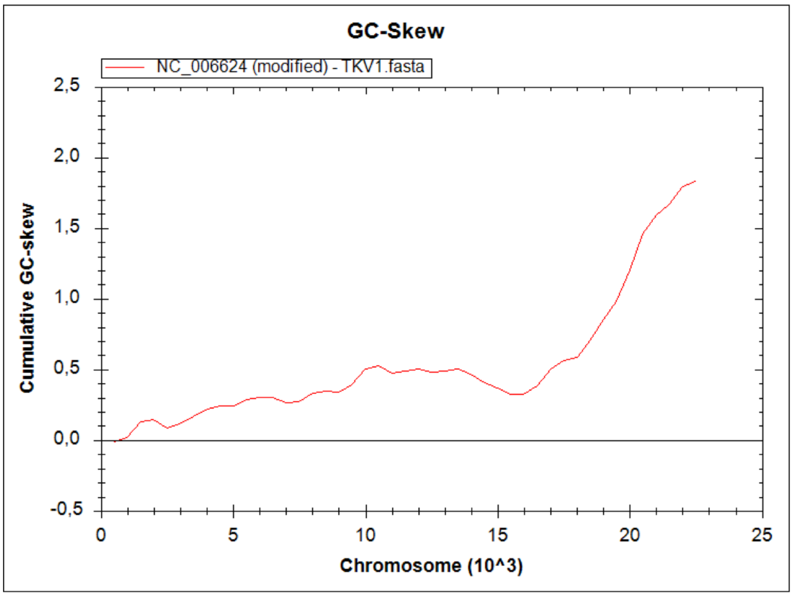

Thermococcus kodakarensis TKV2

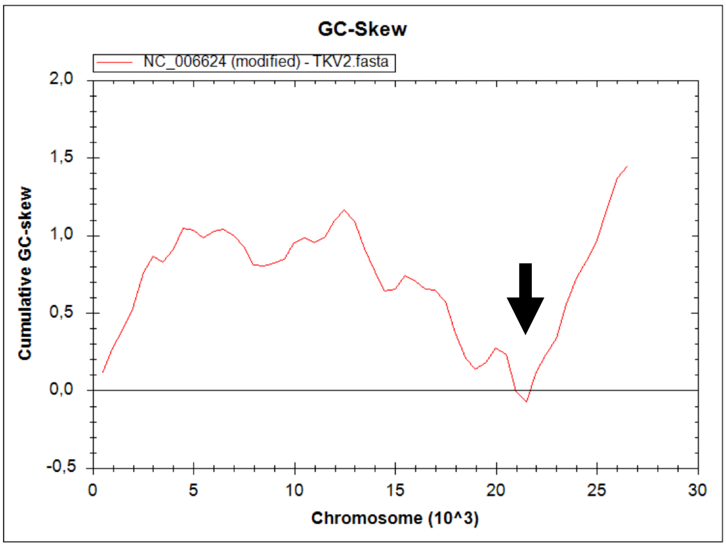

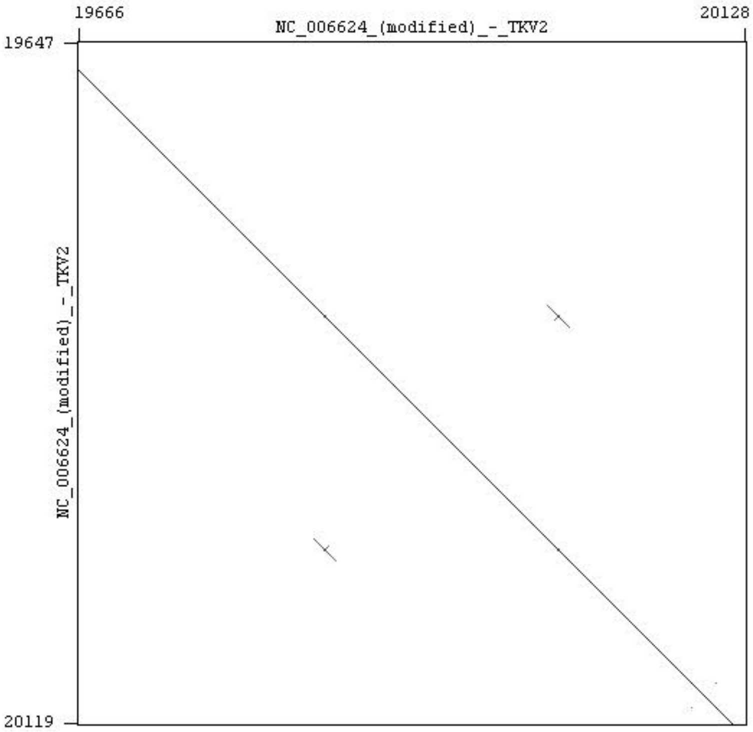

Thermococcus kodakarensis TKV3

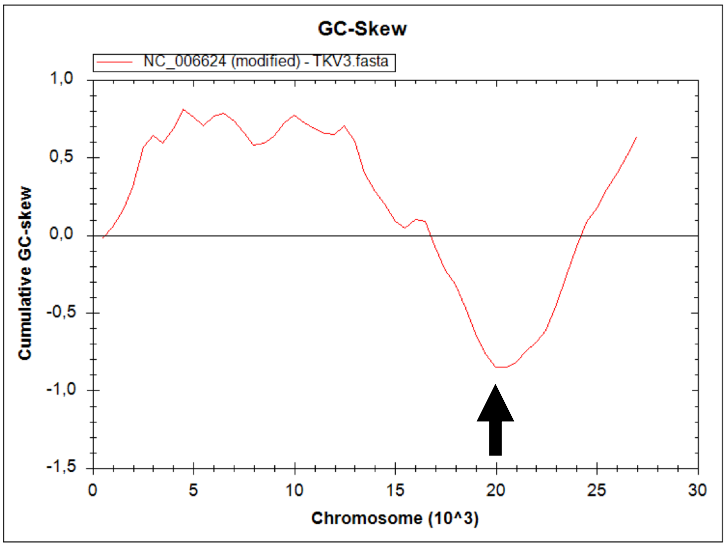

Thermococcus guaymasensis

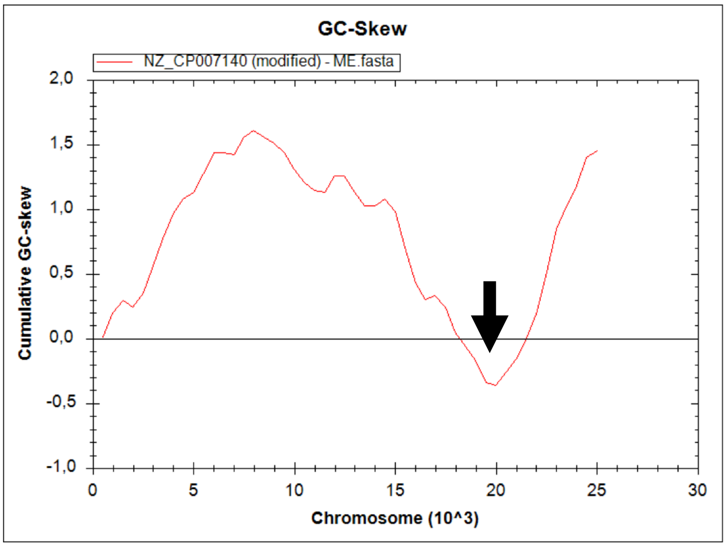

Thermococcus litoralis

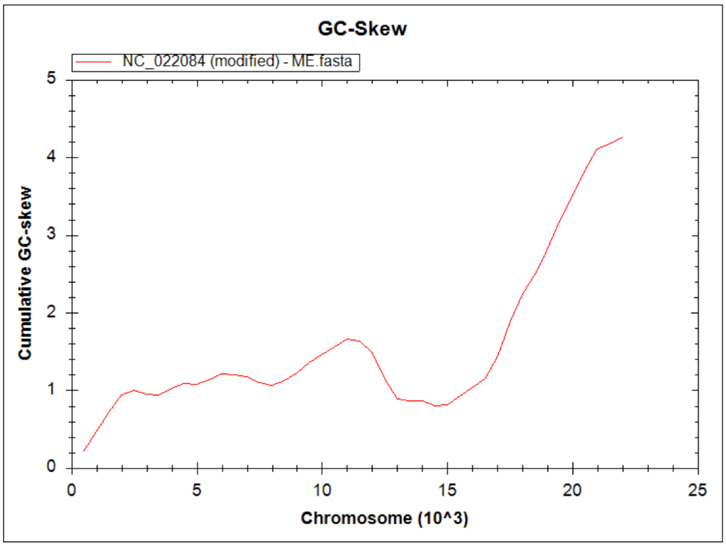

Thermococcus barophilus E1

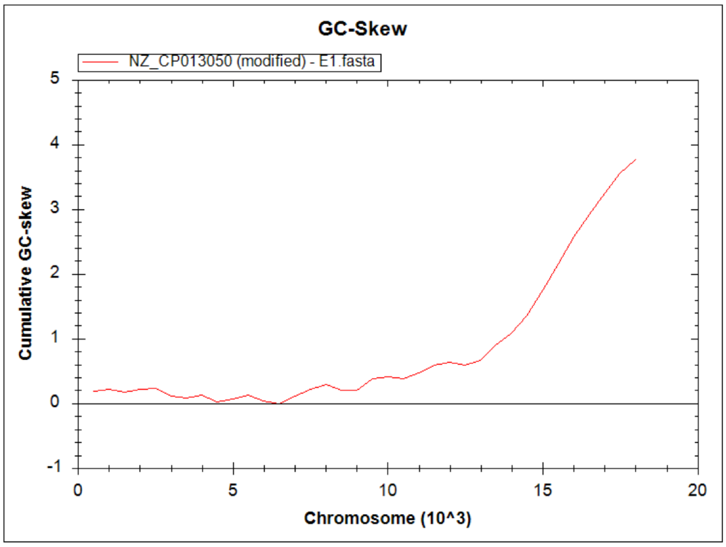

Thermococcus barophilus E2

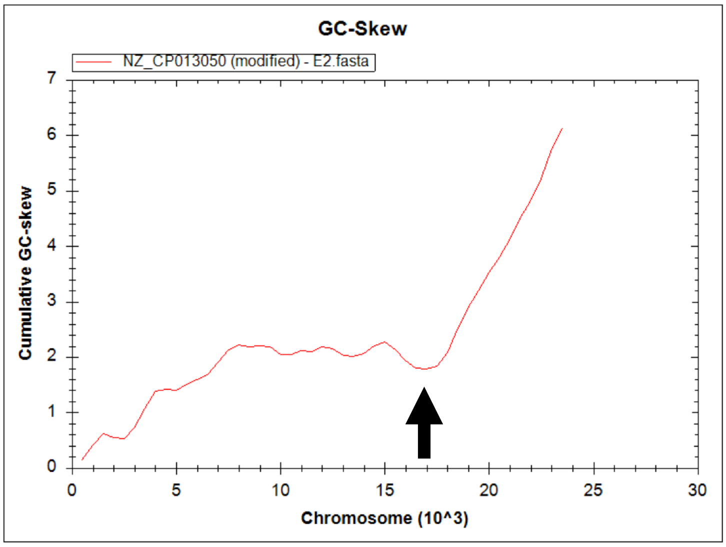

Thermococcus sp. JCM11816

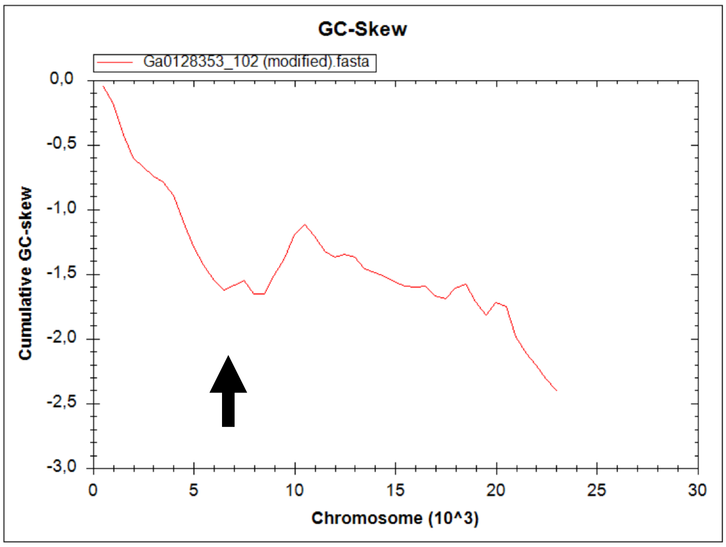

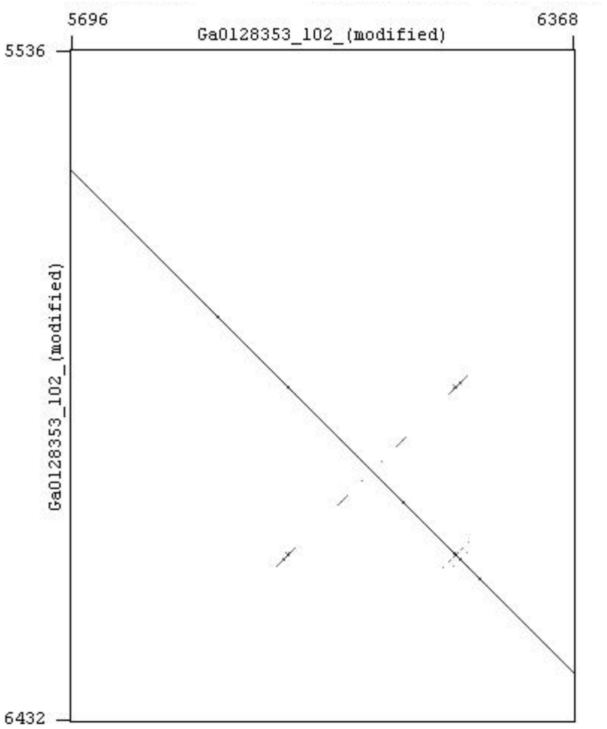

Thermococcus gammatolerans TGV1

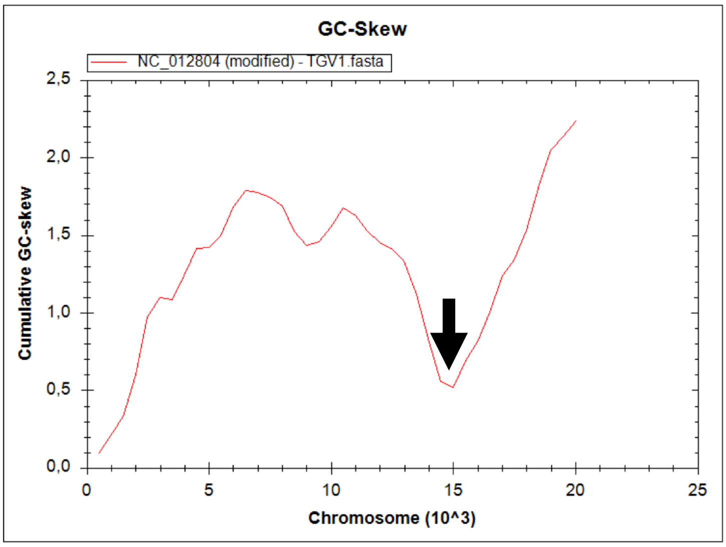

Thermococcus celericrescens

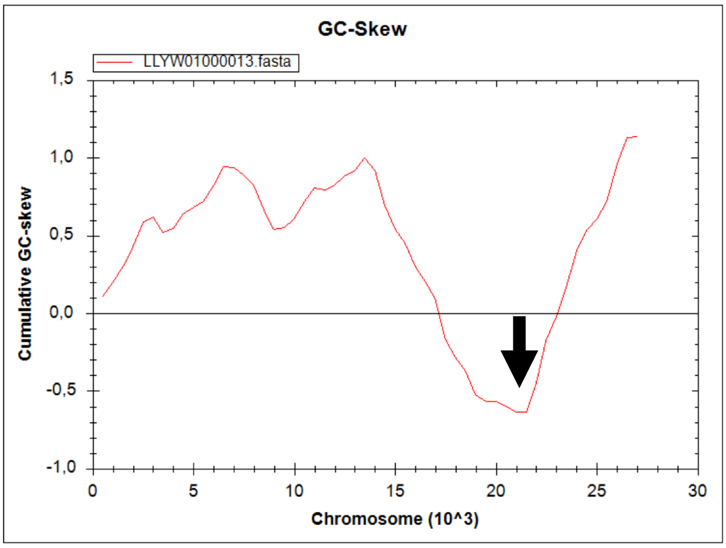

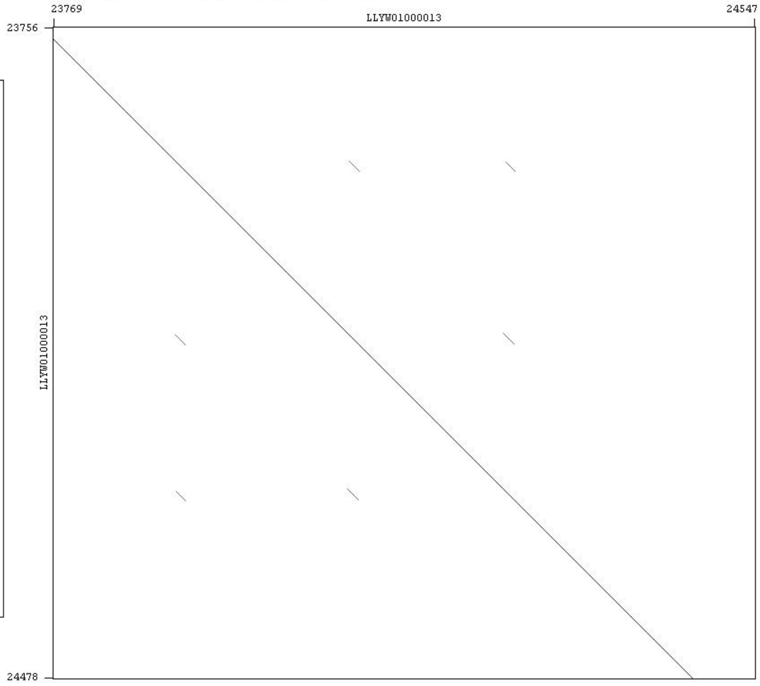

Pyrococcus chitonophagus

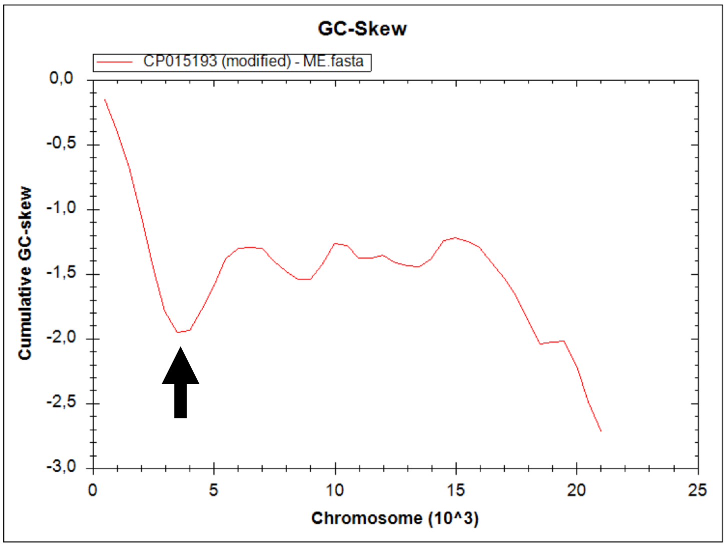

Pyrococcus kukulkanii

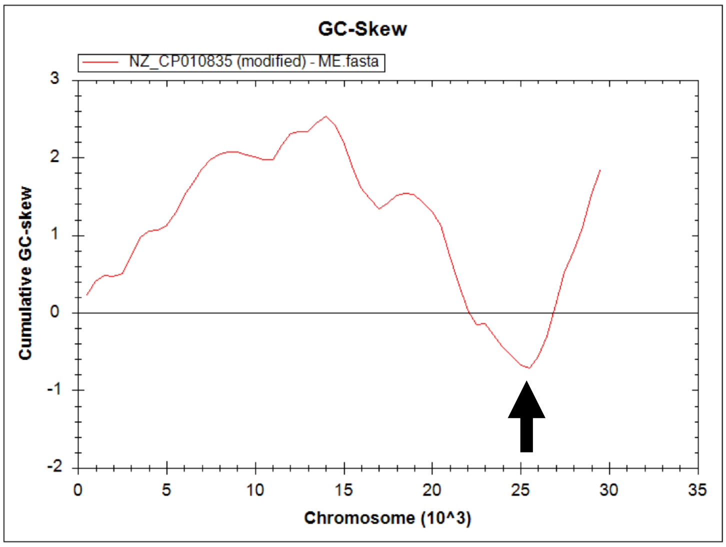

Pyrococcus korikoshii

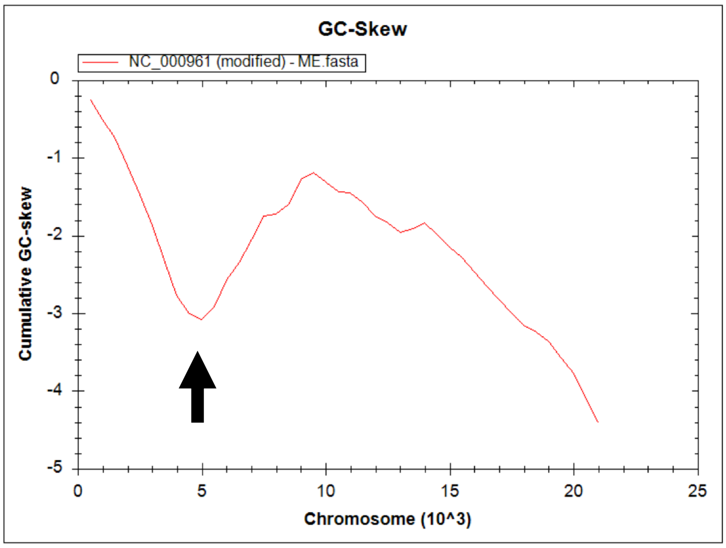

Pyrococcus kukulkanii

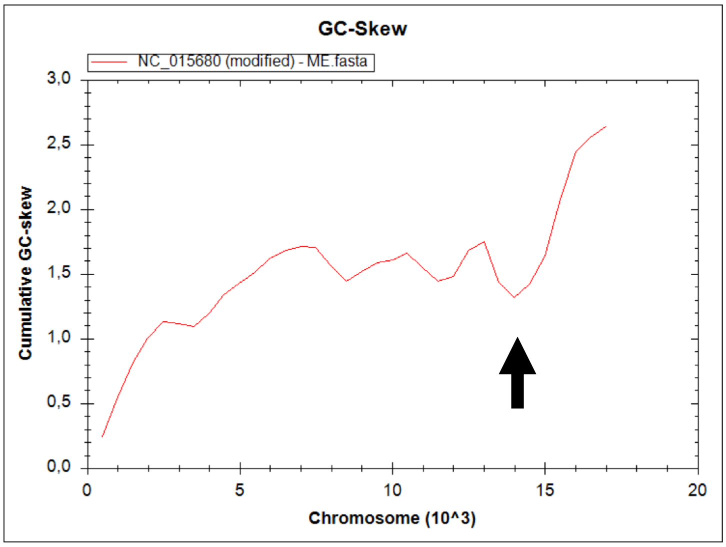

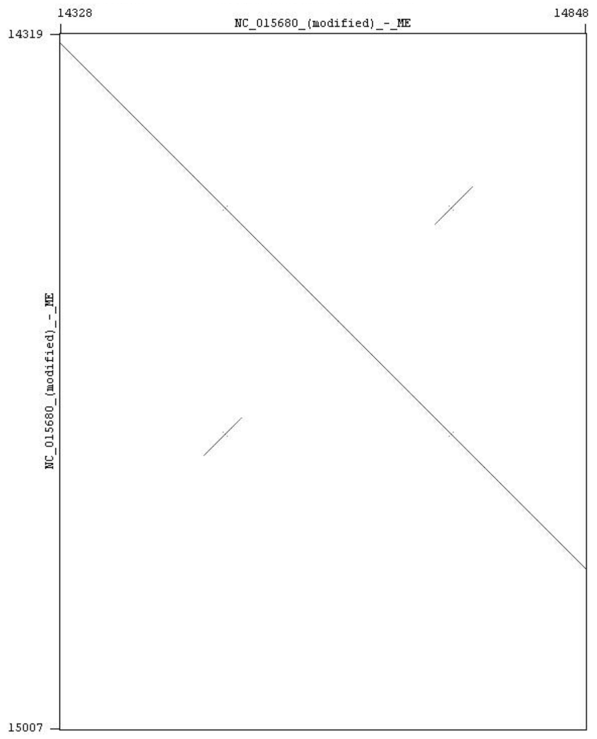

Pyrococcus sp. NA2

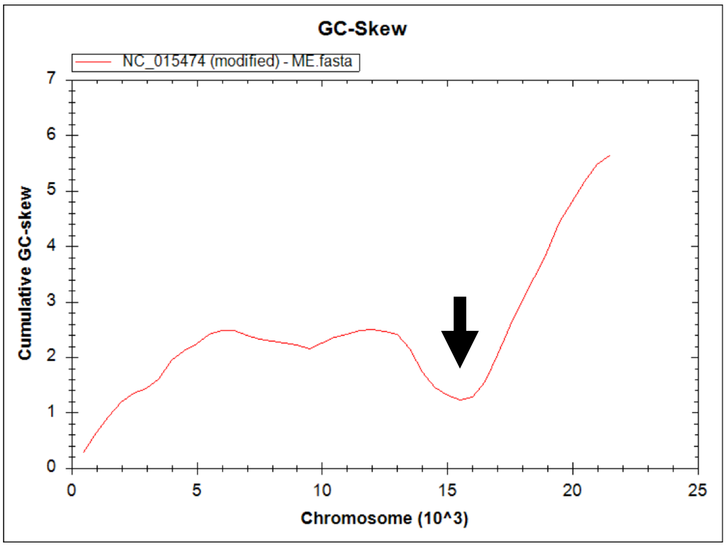

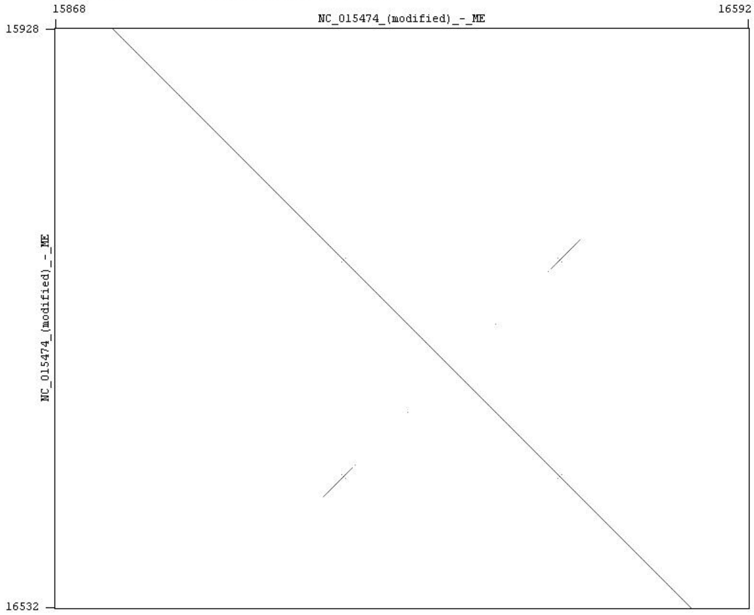

Methanococcus maripaludis MMC6V1

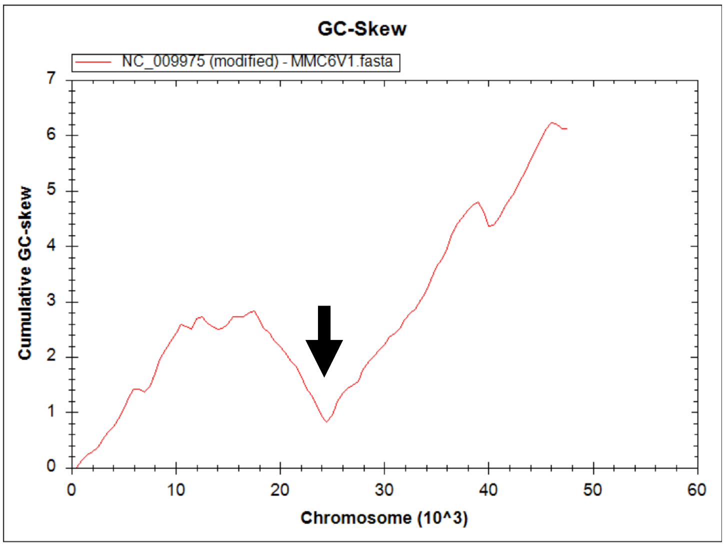

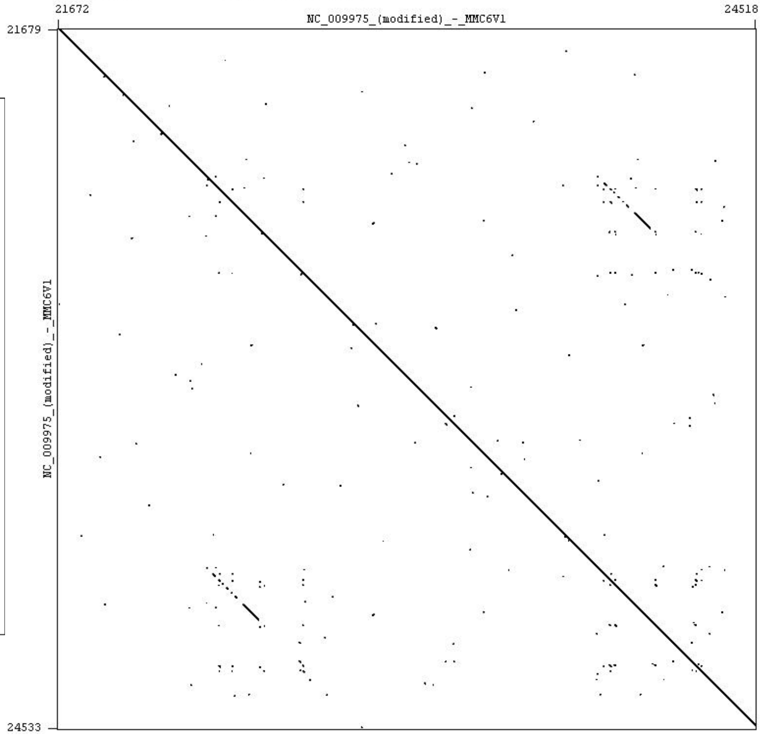

Methanococcus maripaludis MMC7V2

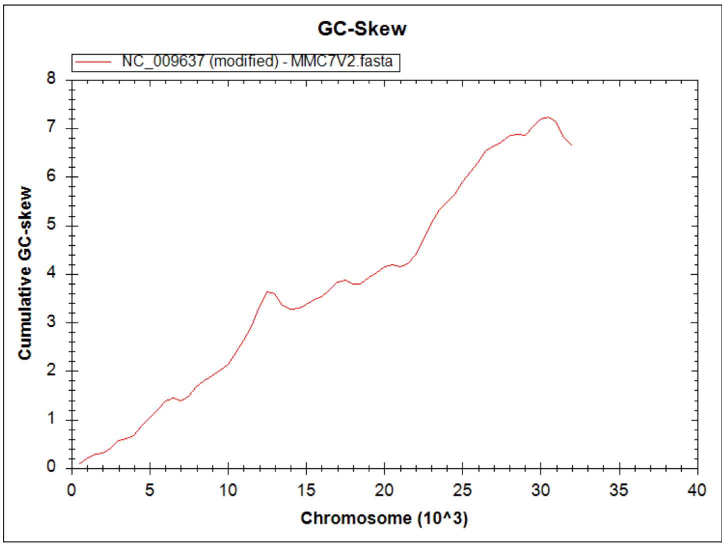

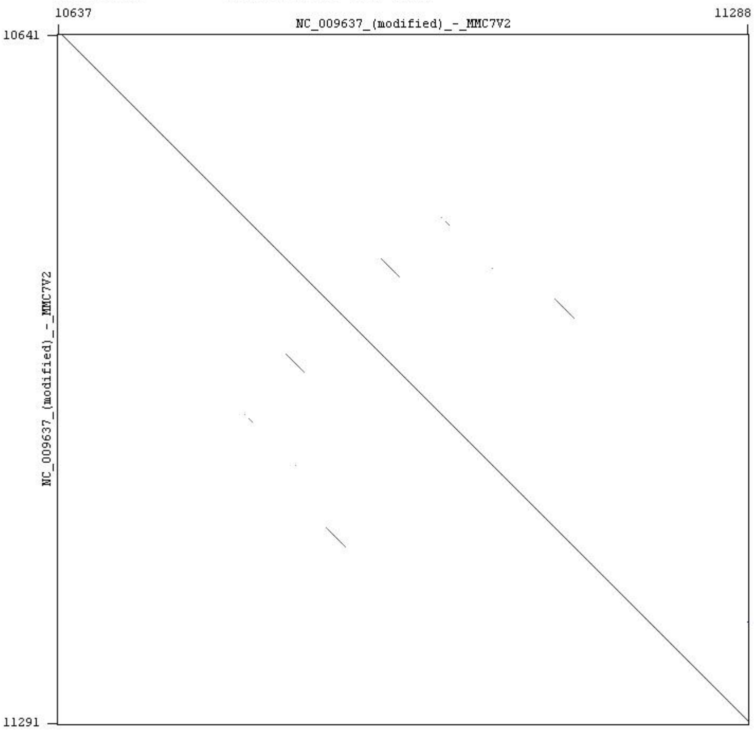

Methanococcus maripaludis S2 MMPV1

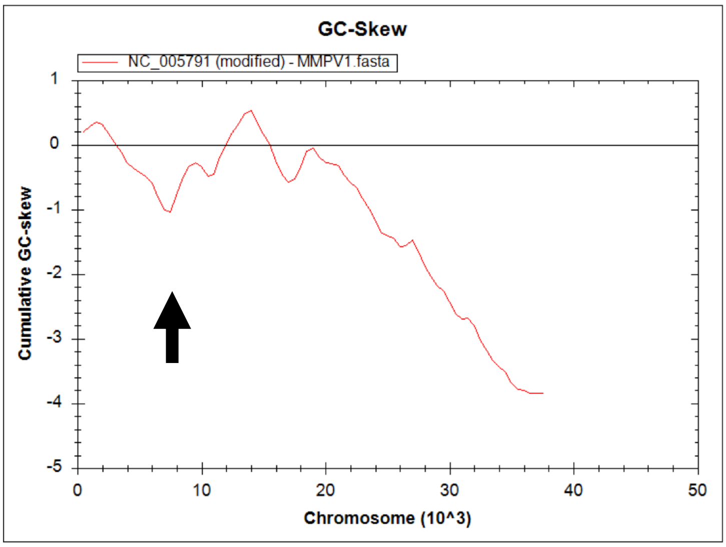

Methanococcus maripaludis KA1

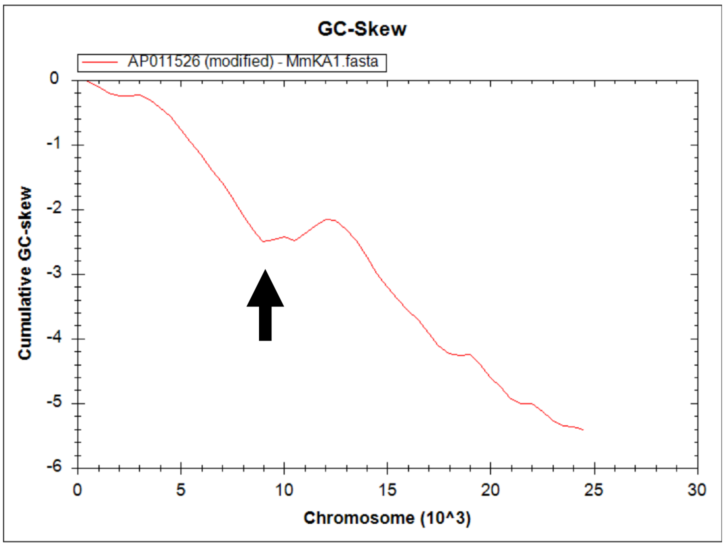

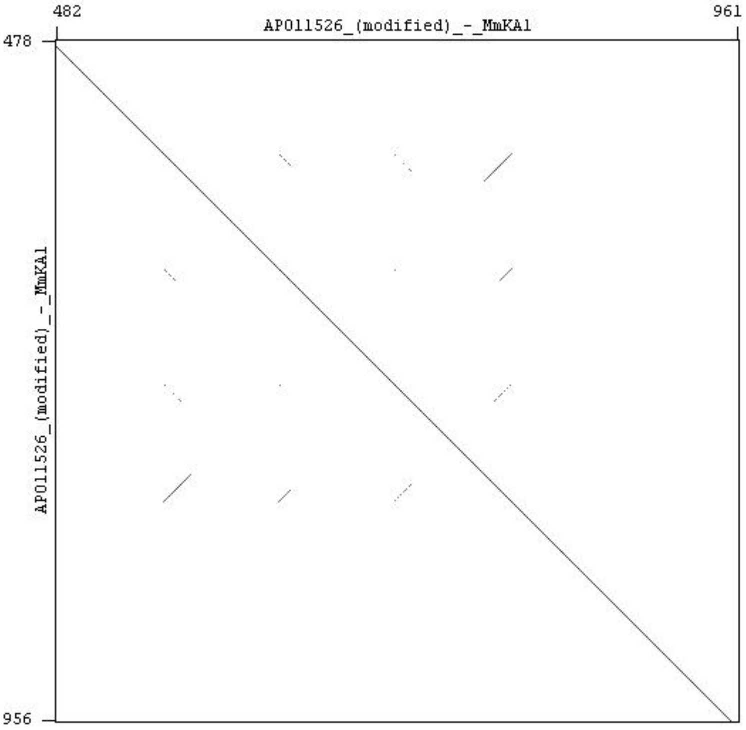

Methanococcus maripaludis OS7

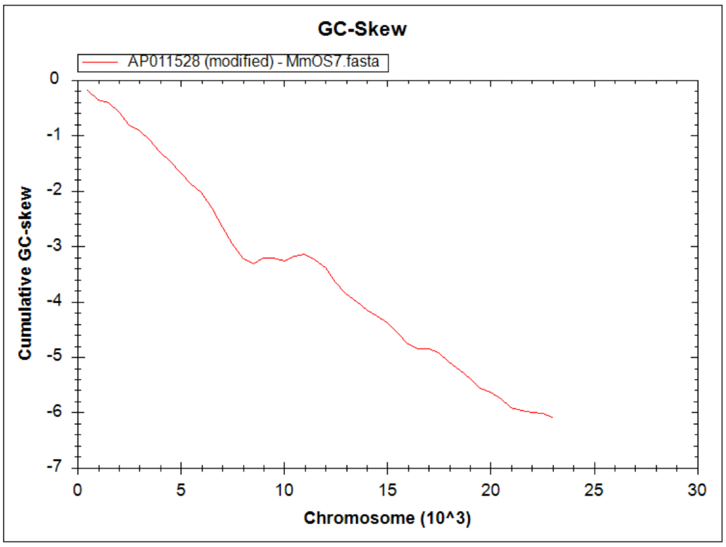

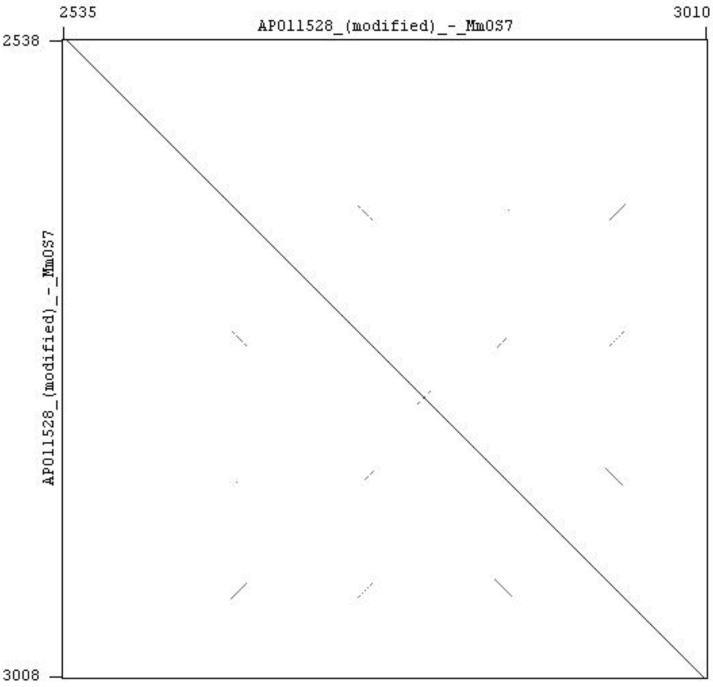

Methanococcus voltae MVV1

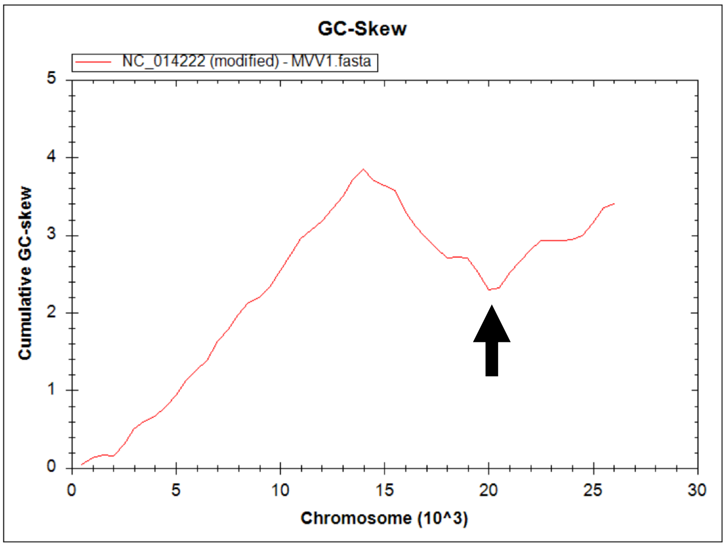

Methanothermococcus thermolithotrophicus

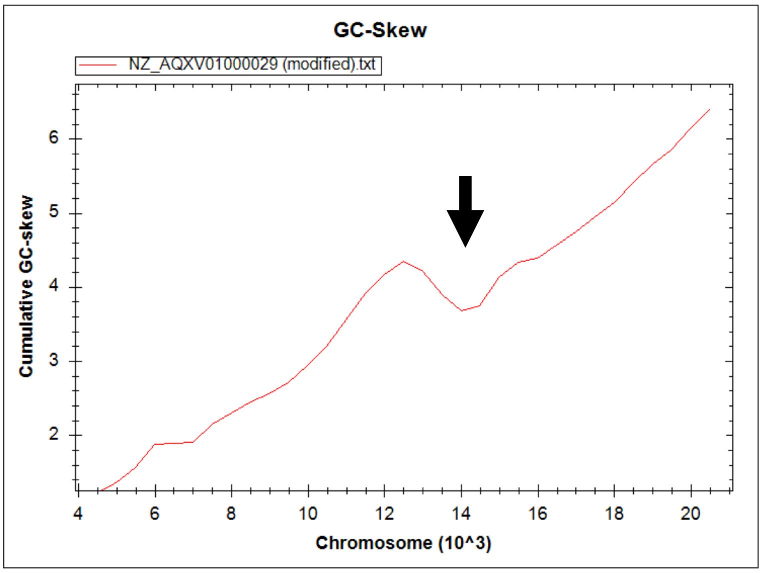

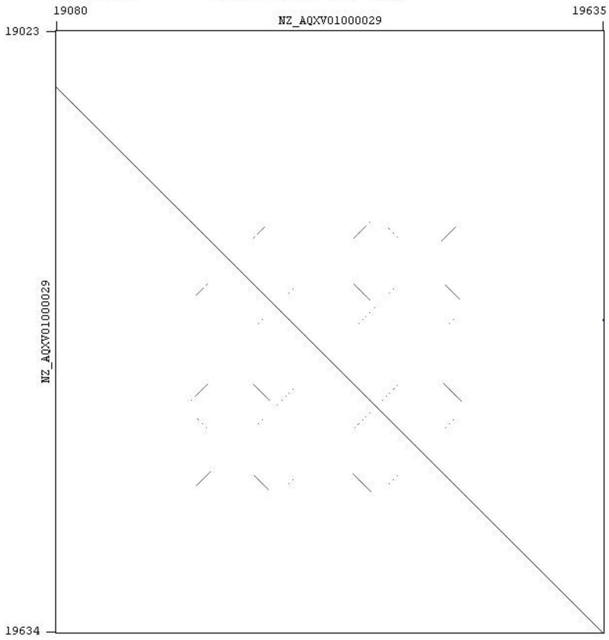

Methanotorris igneus

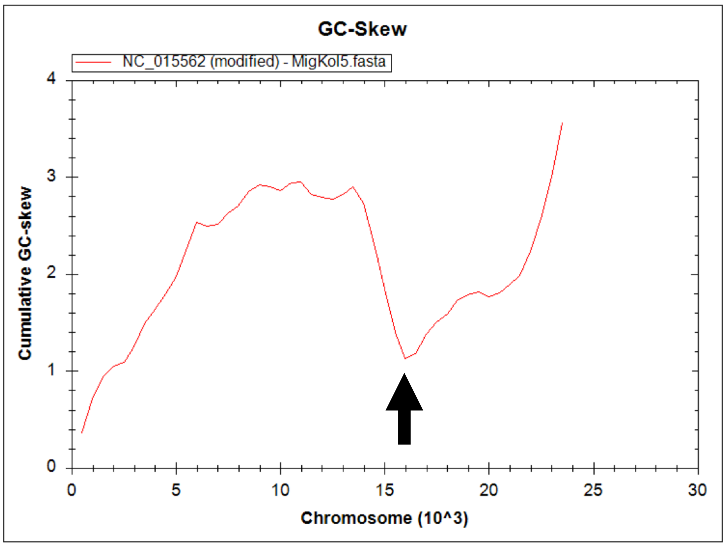

Methanocaldococcus sp. FS406-22

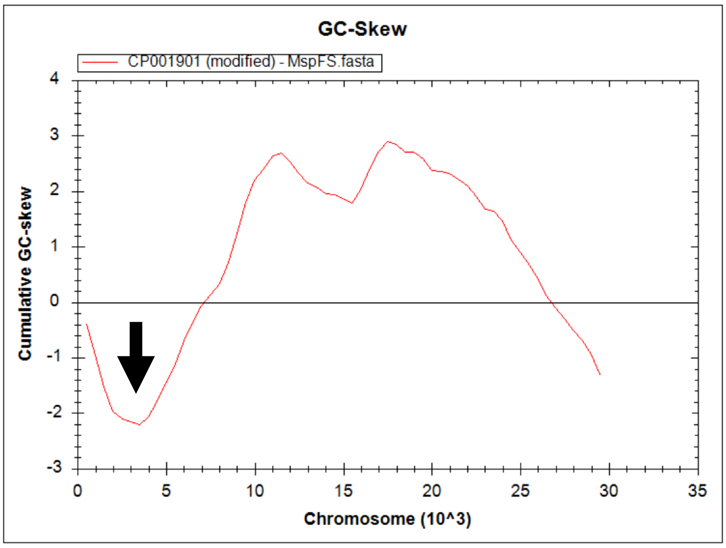

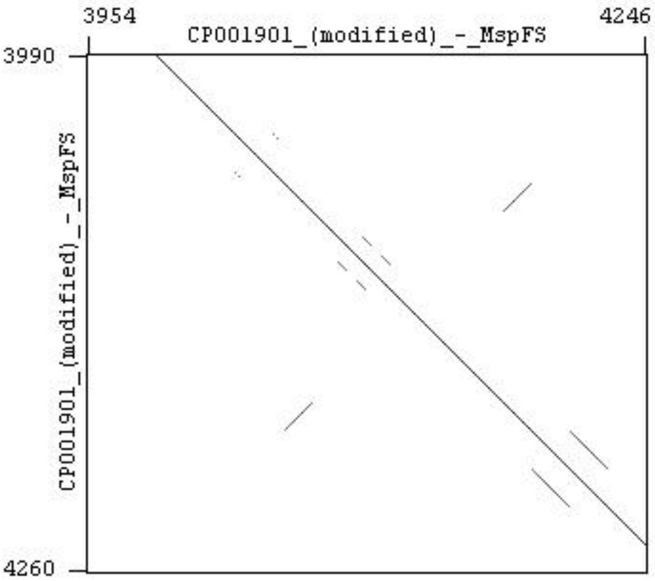

pMEFER01

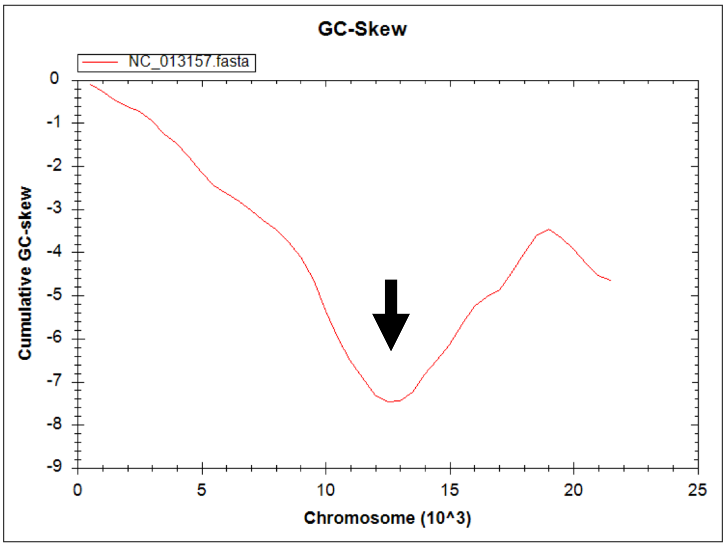

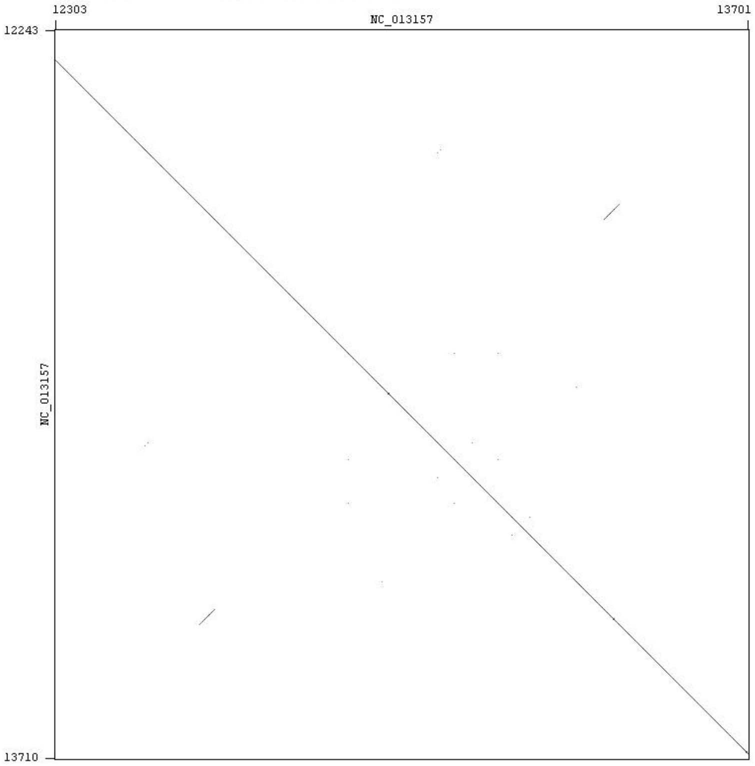

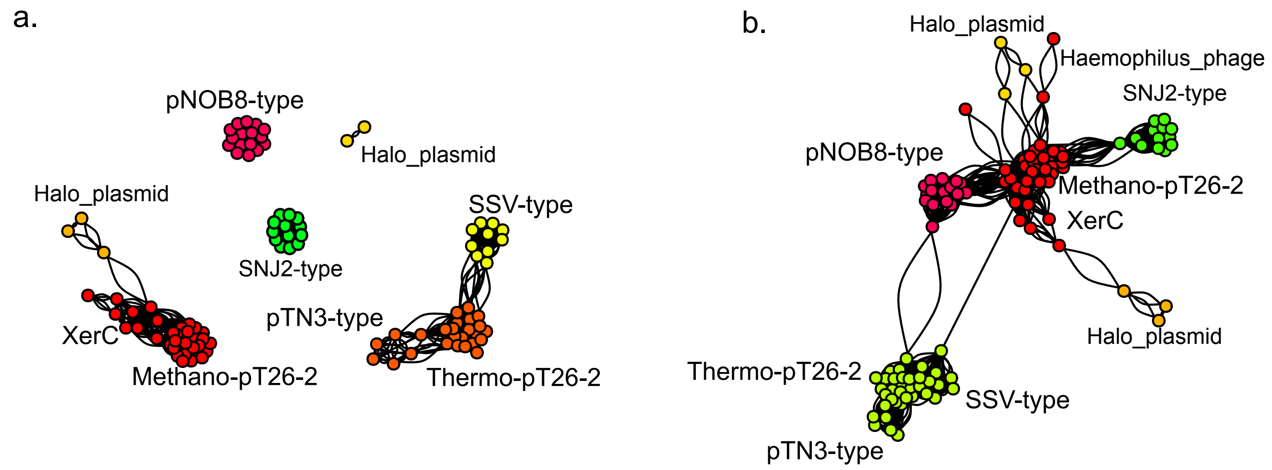

Fig. S10. Integrase similarity network view and corresponding family attribution.
The results of all integrases versus all integrase protein by BlastP (expect >0.001) are represented as a network with two additional different criteria in the limits of similarity in **a** the similarity is >25% among 65% of the protein, and in **b** >25% among 40% of the protein.

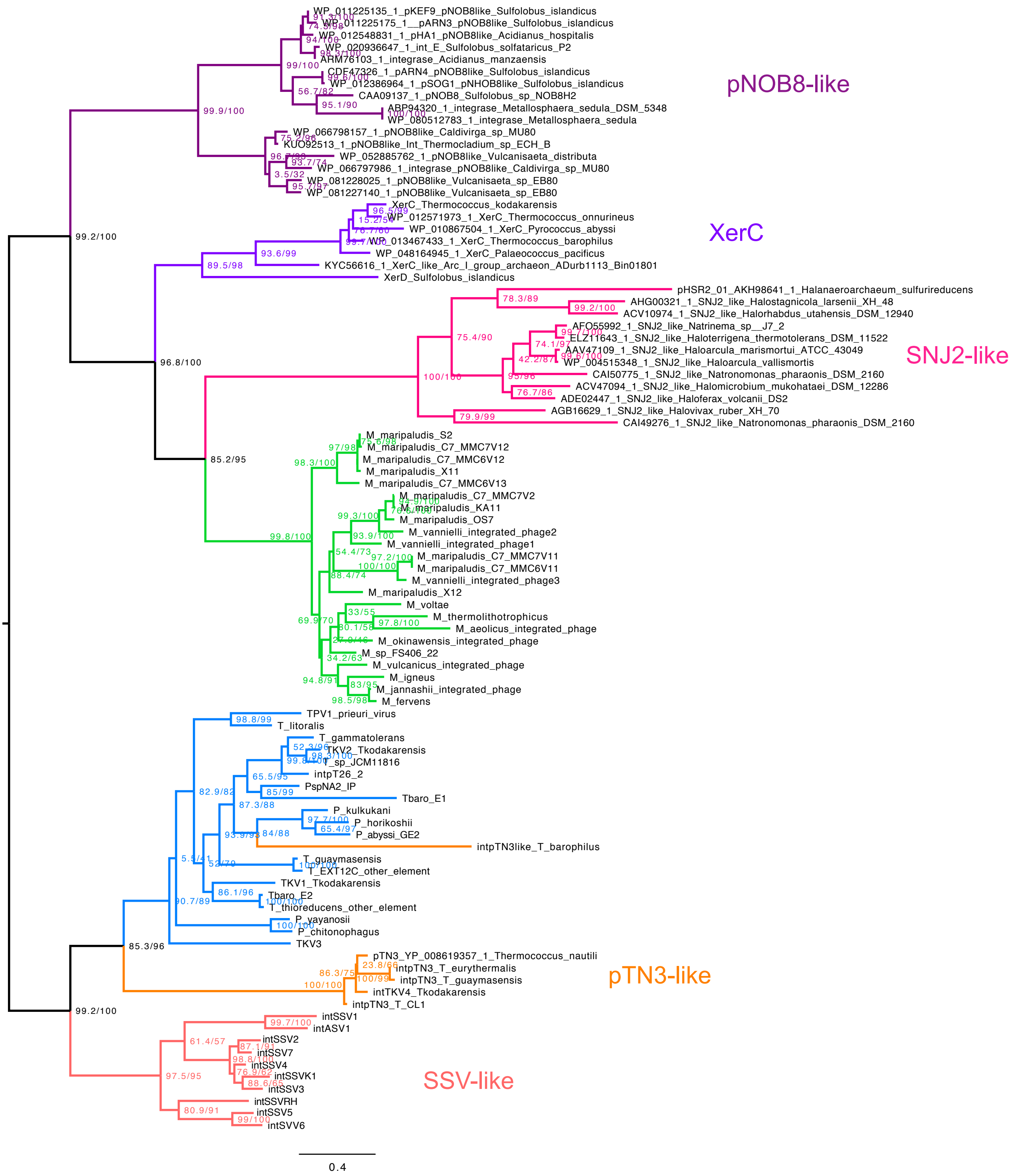

Fig. S11. Two integrases family encoded by plasmid of the pT26-2 family
Maximum Likelihood phylogeny of thepT26-2 encoded integrases protein using several other known integrases family as an outgroup.

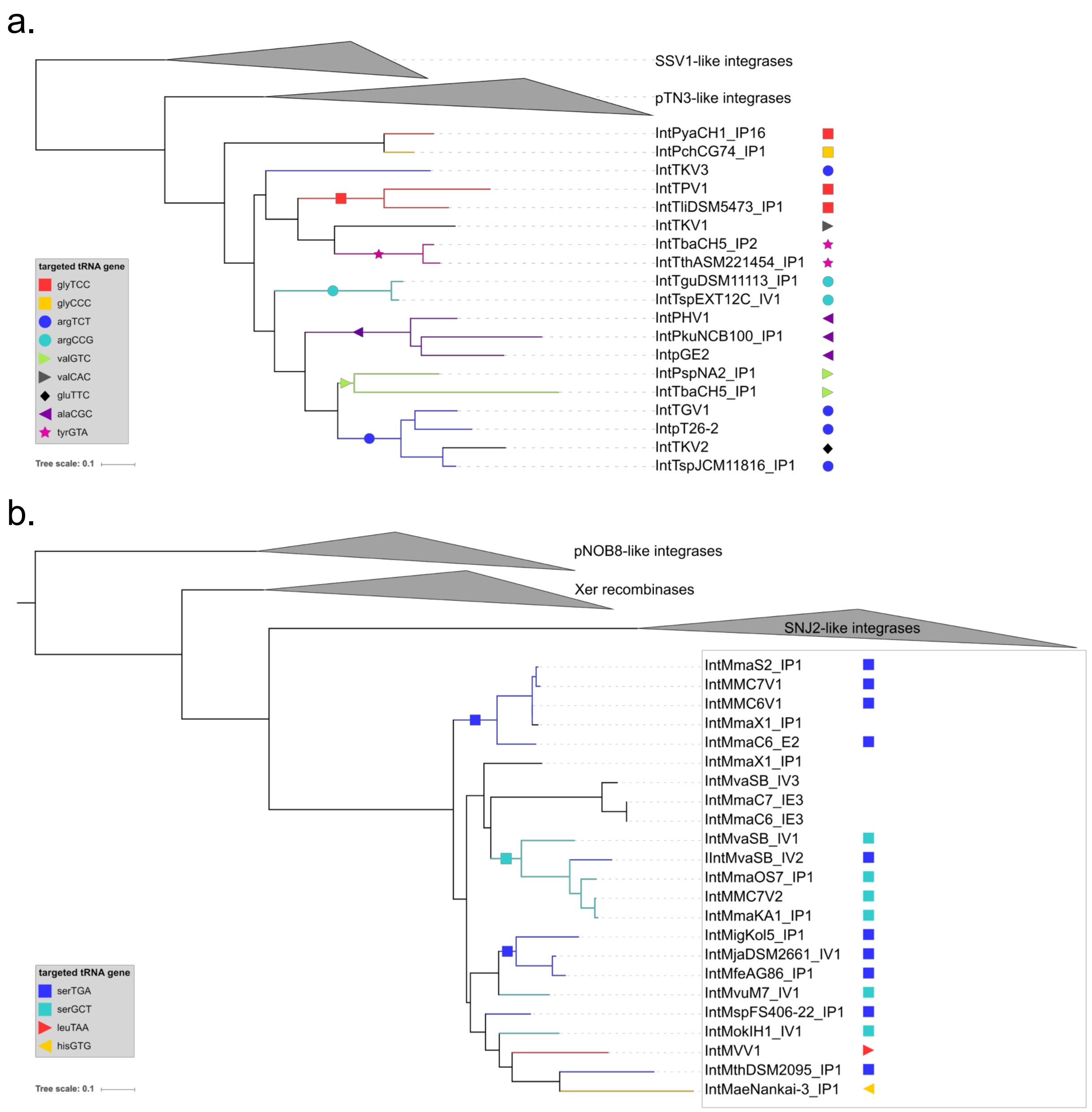

Fig. S12. Integrase phylogeny and targeted tRNA gene for *Thermococcales* (top) and *Methanococcales* (bottom).
Maximum Likelihood tree of integrases proteins (see uncollapsed version in supplementary Fig. S11). The tRNA gene in which the att site is located is indicated by coloured symbols. tRNA corresponding to the same amino-acid but with different anti-codon are indicated by the same symbol but varying colour. Thermococcales integrases target 9 different tRNA genes while Methanococcales integrases target 4 different tRNA genes. Most parsimonious ancestral tRNA gene target is indicated when possible. Integrase homologs found in integrated element that differ from pT26-2 are included.

Table S1. The putative replication origins coordinates in the plasmids of the pT26-2 family. Two complementary methodologies were used to determine the origin of replication : GC-skew and dotplot

Host	element name	ori localisation	methodology
Themococcus sp 26-2	pT26-2	between t26-20p and t26-21p	GC-skew and dotplot
Thermococcus kodakarensis KOD1	TKV1	**Not found**	
Thermococcus kodakarensis KOD1	TKV2	from TK_RS02005 to TK_RS02015	GC-skew and dotplot
Thermococcus kodakarensis KOD1	TKV3	inside TK_RS02910	GC-skew
Thermococcus guayamensis DSM11113		from X802_RS00960 to X802_RS00965	GC-skew
Thermococcus litoralis DSM 5473		**Not found**	
Thermococcus barophilus CH5	E1	**Not found**	
Thermococcus barophilus CH5	E2	from TBCH5V1_RS11215 to TBCH5V1_RS11225	GC-skew
Thermococcus sp. JCM11816 *CONTIG 00002*		between Ga0128353_102299 and Ga0128353_102300	GC-skew and dotplot
Thermococcus gammatolerans EJ3	TGV1	from TGAM_RS03400 to TGAM_RS03405	GC-skew
Thermococcus celericrescens DSM17994 CONTIG 013		from the end of APY94_04115 to APY94_04120 and downstream	GC-skew and dotplot
Pyrococcus chitoniphagus GC74		between A3L04_06365 and A3L04_06370	GC-skew
Pyrorcoccus kukulkanii sp. NCB100		between TQ32_RS02635 and TQ32_RS02645	GC-skew
Pyrococcus horikoshii OT3	PHV1	between PH_RS05530 and PH_RS05540	GC-skew
Pyrococcus yayanosii CH1		between PYCH_RS07010 and PYCH_RS07015	GC-skew and dotplot
Pyrococcus sp. NA2		immediatly downstream of PNA2_RS06700	GC-skew and dotplot
Pyrococcus abyssi GE2	pGE2	In the intergenic region two ORFs upstream the putative replication protein	GC-skew and dotplot
Methanococcus maripaludis C6	MMC6V1	between MMARC6_RS00125 and MMARC6_RS00130	GC-skew and dotplot
Methanococcus maripaludis C7	MMC7V1	**Not found**	
Methanococcus maripaludis C7	MMC7V2	from MMARC7_RS07665 to MMARC7_RS07670	dotplot
Methanococcus maripaludis S2	MMPV1	between MMP_RS03905 and MMP_RS08880	GC-skew
Methanococcus maripaludis KA1		Dotplot: between tRNAser and MMKA1_04820 (or GC-skew between MMKA1_04910 and MMKA1_04970)	
Methanococcus maripaludis OS7		between MMOS7_04780 and MMOS7_04790	dotplot
Methanococcus maripaludis C5		**Not found**	
Methanococcus maripaludis X1		**Not found**	
Methanococcus voltae A3	MVV1	between MVOL_RS08870 and downstream of MVOL_RS07785	GC-skew
Methanothermococcus thermolithotrophicus		Dotplot: between F555_RS08905 and F555_RS0101670<br>GC skew: from F555_RS0101630 to F555_RS0101635	
Methanotorris igneus Kol5		between METIG_RS02530 and METIG_RS02535	GC-skew
Methanocaldococcus sp. FS406-22		between MFS40622_1105 and MFS40622_1106	GC-skew and dotplot
Methanocaldococcus fervens AG86	pMEFER01	between MEFER_RS08100 and MEFER_RS08105	GC-skew and dotplot

Table S2. List of primers

Name	Sequence (5'->3')	Ref
SP-ISC913		This study
ASP-ISC913		This study
SP-pGE2-CDS7	ATGAATACCGGAGTGTTCCTGAAGC	This study
ASP-pGE2-CDS7	AACGATGGCGTAACTTACGGTAAGA	This study
SP-pGE2-CDS29	TTGCTGCGTTTAGAATTAGCTCGTT	This study
ASP-pGE2-CDS29	TGGGTTGGGAGTACACCATAAAGAA	This study
Arc344F	ACGGGGYGCAGCAGGCGCGA	Raskin et al., 1994
Uni516R	GTDTTACCGCGGCKGCTGRCA	Takai & Horikoshi 2000

Raskin L, Stromley JM, Rittmann BE, Stahl DA (1994) Group-specific 16S rRNA hybridization probes to describe natural communities of methanogens. *Appl Environ Microbiol* 60: 1232–1240.
Takai & Horikoshi (2000) Rapid detection and quantification of members of the archaeal community by quantitative PCR using fluorogenic probes. *Appl Environ Microbiol* 66 : 5066-5072
